# Transetherification of 2,4-dimethoxynitrobenzene by aromatic nucleophilic substitution

**DOI:** 10.1371/journal.pone.0183575

**Published:** 2017-08-23

**Authors:** Jiho Song, Hae Ju Kang, Jung Wuk Lee, Michelle A. Wenas, Seung Hwarn Jeong, Taeho Lee, Kyungsoo Oh, Kyung Hoon Min

**Affiliations:** 1 College of Pharmacy, Chung-Ang University, Seoul, Republic of Korea; 2 College of Pharmacy, Kyungpook National University, Daegu, Republic of Korea; University of Bradford, UNITED KINGDOM

## Abstract

In view of the few reports concerning aromatic nucleophilic substitution reactions featuring an alkoxy group as a leaving group, the aromatic nucleophilic substitution of 2,4-dimethoxynitrobenzene was investigated with a bulky *t*-butoxide nucleophile under microwave irradiation. The transetherification of 2,4-dimethoxynitrobenezene with sodium *t*-butoxide under specific conditions, namely for 20 min at 110°C in 10% dimethoxyethane in toluene, afforded the desired product in 87% yield with exclusive *ortho*-selectivity. A variety of reaction conditions were screened to obtain the maximum yield. The aromatic nucleophilic substitution of 2,4-dimethoxynitrobenzene with *t*-butoxide should be carried out under controlled conditions in order to avoid the formation of byproducts, unlike that of dihalogenated activated benzenes. Among the formed byproducts, a major compound was elucidated as 2,4-dimethoxy-*N*-(5-methoxy-2-nitrophenyl)aniline by X-ray crystallography.

## Introduction

**Fig 1:**
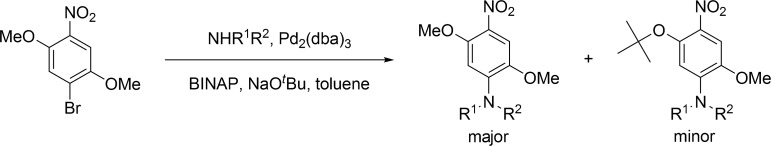
Identification of a byproduct from a palladium catalyzed aryl amination reaction.

The aromatic nucleophilic substitution (S_N_Ar) reaction is a well-known method to conveniently introduce a variety of substituents onto activated aromatics, which has been investigated for aryl halides with strong electron-withdrawing groups such as nitro and ester groups for about a century [1]. S_N_Ar reactions are still extensively utilized, especially in the pharmaceutical industry [2, 3], despite the development of useful transition metal catalyzed cross-coupling reactions. The regioselectivity of the substitution at *ortho* versus *para* positions induced by an electron-withdrawing group has been one of the main issues in S_N_Ar reactions. Although the reactions could be carefully controlled to achieve the desired regioselectivity in case of substrates having two identical leaving groups, a few studies have reported the reaction conditions necessary to attain high regioselectivity in the S_N_Ar reactions for substrates activated with common dihalogens using a variety of nucleophiles [4–9]. Wendt et al. described the solvent effect on the *ortho*-selectivity in the S_N_Ar reactions of 2,4-dihaloaromatic compounds [5]. Synthana et al. demonstrated that nonpolar solvents play a key role in achieving high *o*-selectivity in the S_N_Ar reactions of 2,4-difluoronitrobenzene [6]. However, the leaving groups in most S_N_Ar reactions have been mainly limited to halogens, although the nitro group has also been reported in a few exceptional cases. Few limited examples have shown that alkoxy groups could also act as leaving groups in S_N_Ar reactions [10, 11]. In this study, we observed the unexpected formation of a *t*-butoxide-substituted byproduct as a minor product during a palladium catalyzed aryl amination reaction, as shown in Fig 1. To the best of our knowledge, *ortho*-selective S_N_Ar reaction of a methoxy group as a leaving group has never been reported. Herein, the *ortho*-selective transetherification of 2,4-dimethoxynitrobenzene by an S_N_Ar reaction with a *t*-butoxide bulky nucleophile was explored.

## Materials and methods

### General methods

Unless otherwise noted, reagents and solvents were purchased from commercial suppliers (Sigma-Aldrich, Combi-Blocks, TCI, Alfa Aesar, or samchun chemicals), and solid reagents were used without further purification. Purchased liquid reagents and solvents were dried over 4Å molecular sieves or distilled before use. Microwave reactions were performed in sealed glass vial using Anton Paar Monowave 300, a specially designed microwave reactor for chemical research. Flash column chromatography was conducted using silica gel (ZEOCHEM, ZEOprep 60, 40–63 μm) manually or using prepacked flash column (WELUX) on Combi-Flash (Teledyne ISCO). ^1^H NMR spectra were recorded on Varian Germini 2000 (300 MHz) or Varian VNS (600 MHz), and the ^13^C NMR spectra were recorded on Varian VNS (150 MHz). Chemical shifts were reported in parts per million (ppm, *δ*) downfield from tetramethylsilane (TMS), and coupling constants *J* were reported in hertz (Hz). HPLC analysis was conducted on Waters 1525 binary HPLC pump system equipped with a Waters 2998 photodiode array detector using SunFire™ C18 column (4.6 × 150 mm, 5μm particle size). Eluent system was 0.1% formic acid in water: Acetonitrile = 80: 20 to 0: 100 over 15 min at a flow rate = 1.2 mL/min. HRMS analyses were carried out on a DIONEX Ultimate 3000 system (LC) and Thermo Scientific Q-Exactive system (MS) using a Thermo Scientific Hypersil GOLD C18 (2.1mm × 50 mm, 1.9 μm). Infrared spectra were recorded on Thermo Scientific Nicolet 6700 FT-IR spectrometer.

### General experimental procedure for Tables 1–4.

A mixture of 2,4-dimethoxy-1-nitrobenzene (55 mg, 0.3 mmol), *tert*-butoxide salt, and additive in solvent (1.5 mL) was reacted under microwave irradiation. The reaction mixture was diluted with DCM, filtered through Celite and concentrated under reduce pressure. The crude material was subjected to HPLC analysis to determine the conversion.

**Table 1:** Optimization of reaction temperature and time.*

Entry	Temperature (°C)	Time (min)	Yield (%)**		
			2	3	1
1	100	10	13	0	85
2		20	25	0	73
3		30	28	0	68
4		40	30	29	0
5	110	10	21	0	72
6		20	36	0	64
7		30	48	0	49
8	120	10	31	0	62
9		20	31	28	0
10	130	10	29	29	0
11	140	10	20	39	0
12	150	10	11	39	0
13	160	10	0	44	0

*Reactions were conducted with 2,4-dimethoxy-1-nitrobenzene (0.3 mmol) and NaO^*t*^Bu (0.9 mmol) in toluene (1.5 mL) under microwave irradiation.

** Determined by HPLC analysis from each standard curve.

**Table 2:** Optimization of the amount of nucleophile and the effect of the counterion of *t*-butoxide.*

Entry	Nucleophile (equiv.)	Temperature (°C)	Time (min)	Yield (%)**		
				2	3	1
1	NaO^*t*^Bu (1)	110	20	14	0	84
2	NaO^*t*^Bu (1)	110	30	16	0	83
3	NaO^*t*^Bu (1.5)	110	20	15	0	65
4	NaO^*t*^Bu (1.5)	110	30	32	0	40
5	NaO^*t*^Bu (3)	110	20	36	0	64
6	NaO^*t*^Bu (3)	110	30	48	0	49
7	NaO^*t*^Bu (5)	110	20	54	0	42
8	NaO^*t*^Bu (5)	110	30	66	1	29
9	NaO^*t*^Bu (7)	110	20	72	0	27
10	NaO^*t*^Bu (7)	110	30	49	9	0
11	NaO^*t*^Bu (10)	110	20	28	18	2
12	LiO^*t*^Bu (7)	110	20	0	0	99
13	LiO^*t*^Bu (7)	120	20	0	0	99
14	LiO^*t*^Bu (7)	130	20	2	0	92
15	LiO^*t*^Bu (7)	140	20	3	0	89
16	LiO^*t*^Bu (7)	150	20	1	5	80
17	LiO^*t*^Bu (7)	160	20	2	16	46
18	KO^*t*^Bu (7)	60	20	39	0	8
19	KO^*t*^Bu (7)	90	20	0	3	0
20	KO^*t*^Bu (7)	110	20	0	2	0

*Reactions were conducted with 2,4-dimethoxy-1-nitrobenzene (0.3 mmol) and a base in toluene (1.5 mL) under microwave irradiation.

^**^ Determined by HPLC analysis.

**Table 3:** Optimization of the reaction concentration.*

Entry	concentration (M)	Yield (%)**		
		2	3	1
1	0.05	28	0	67
2	0.1	45	0	54
3	0.2	72	0	27
4	0.5	22	22	1

^*^Reactions were conducted with 2,4-dimethoxy-1-nitrobenzene (0.3 mmol) and NaO^*t*^Bu (2.1 mmol) in toluene under microwave irradiation.

** Determined by HPLC.

**Table 4:** Effect of the solvent.*

Entry	Solvent	Yield (%)**		
		2	3	1
1	Toluene	72	0	27
2	Benzene	23	16	0
3	Xylene	62	0	18
4	1,4-Dioxane	0	32	0
5	1,2-Dichloroethane (DCE)	0	0	100
6	Hexamethylphosphorous triamide (HMPT)	28	4	1
7	Tetrahydrofuran (THF)	0	0	0
8	*N*-Methyl-2-pyrrolidone (NMP)	0	0	0
9	Dimethylformamide (DMF)	0	0	0
10	Dimethoxyethane (DME)	0	32	0
11	*tert*-BuOH	0	0	0
12	Cyclohexanol	0	0	0
13	Toluene (9): DME (1)	87	0	10
14	Toluene (5): DME (1)	34	14	0
15	Toluene (2): DME (1)	0	28	0
16	Toluene (9): Diethylether (1)	23	19	0

^*^Reactions were conducted with 2,4-dimethoxy-1-nitrobenzene (0.3 mmol) and NaO^*t*^Bu (2.1 mmol) in a solvent (1.5 mL) under microwave irradiation.

** Determined by HPLC analysis.

#### Spectroscopic data of products

2-*tert*-Butoxy-4-methoxy-1-nitrobenzene (**2**). ^1^H NMR (600 MHz, CD_3_CN) *δ* 7.80 (d, *J* = 8.4 Hz, 1H), 6.71–6.74 (m, 2H), 3.85 (s, 3H), 1.39 (s, 9H); ^13^C NMR (150 Hz, CD_3_CN) *δ* 164.4, 152.3, 139.7, 127.8, 110.7, 109.6, 83.8, 56.8, 29.0; HRMS (ESI): m/z calcd for C_11_H_14_NO_4_ [M—H]^-^, 224.0928, found 224.0917; IR (Neat) ν (cm^-1^): 2980, 2939, 1603, 1517, 1484, 1443, 1392, 1368, 1347, 1295, 1275, 1244, 1207, 1152, 1097, 1029, 974, 867, 845, 821.

2,4-dimethoxy-N-(5-methoxy-2-nitrophenyl)aniline (**3**). ^1^H NMR (600 MHz, CD_3_CN) *δ* 9.39 (s, 1H), 8.10 (d, *J* = 9.6 Hz, 1H), 7.26 (d, *J* = 8.4 Hz, 1H), 6.67 (d, *J* = 3 Hz, 1H), 6.58 (dd, *J* = 8.4, 1.5 Hz, 1H), 6.31–6.33 (m, 1H), 6.26 (d, *J* = 2.4 Hz, 1H), 3.82 (s, 3H), 3,80 (s, 3H), 3.69 (s, 3H); ^13^C NMR (150 MHz, CD_3_CN) *δ* 166.7, 160.2, 155.9, 147.6, 129.6, 128.0, 128.0, 120.9, 107.1, 105.8, 110.5, 97.9, 56.5, 56.4, 56.2; HRMS (ESI): m/z calcd for C_15_H_17_N_2_O_5_ [M + H]^+^, 305.1132 found 305.1132; IR (Neat) ν (cm^-1^): 3340, 2938, 2837, 1619, 1579, 1509, 1493, 1461, 1415, 1337, 1306, 1262, 1233, 1209, 1160, 1084, 1033, 830.

#### Synthesis of nitrobenzene substrates

4-Bromo-2-methoxy-1-nitrobenzene (**4e**). To a solution of 4-Bromo-2-fluoro-1-nitrobenzene (400 mg, 2.0 mmol) in methanol (5 mL) was added sodium methoxide 5.0 M in methanol (480 μL, 2.4 mmol) and then stirred at room temperature for 15 h. The reaction mixture was diluted with ethyl acetate, washed with water and brine, and dried over anhydrous MgSO_4_. The crude material was purified by silica gel chromatography (6% ethyl acetate in hexanes) to give **4e** (380 mg, 73%). ^1^H NMR (600 MHz, Acetone-*d*_6_) δ 7.80 (d, *J* = 8.6 Hz, 1H), 7.53 (d, *J* = 1.9 Hz, 1H), 7.31 (dd, *J* = 8.6, 1.9 Hz, 1H), 4.04 (s, 3H); ^13^C NMR (150 MHz, Acetone*-d*_*6*_) *δ* 154.1, 139.9, 128.4, 127.3, 124.3, 118.3, 57.6; IR (Neat) ν (cm^-1^): 3108, 2990, 2957, 2855, 1611, 1567, 1523, 1441, 1396, 1344, 1304, 1258, 1189, 1005, 872, 858. ^1^H NMR data correspond with those reported in the literature [12].

1-Bromo-2,5-dimethoxy-4-nitrobenzene (**4f**). To a solution of 1-bromo-2,5-dimethoxy (4.31 mL, 30 mmol) in acetic acid (10 mL) was added nitric acid (3.12 mL, 7.5 mmol) over a 5 min period (dropwise). The solution was stirred at room temperature for 30 min and then quenched with water. The yellow solids were filtered off and washed with water. The precipitate was dried in a vacuum to give **4f** (7.76 g, 99%). ^1^H NMR (600 MHz, Acetone-*d*_6_) δ 7.59 (s, 1H), 7.56 (s, 1H), 3.98 (s, 3H), 3.96 (s, 3H); ^13^C NMR (150 MHz, Acetone*-d*_*6*_) *δ* 150.5, 147.7, 139.8, 120.2, 117.9, 109.3, 57.9, 57.5; IR (Neat) ν (cm^-1^): 1508, 1490, 1457, 1437, 1376, 1339, 1217, 1014, 865, 776, 749, 669. ^1^H NMR data correspond with those reported in the literature [13].

4-*tert*-Butyl-2-methoxy-1-nitrobenzene (**4g**). The nitration of 5-*tert*-butylphenol was performed according to the procedure reported by Buckingham *et al*. was followed [14]. To a solution of 5-*tert-*butylphenol (1.5 g, 10.0 mmol) in acetic acid (7 mL) was added nitric acid (0.5 mL, 10.0 mmol) over a 5 min period (dropwise). The solution was stirred at 0°C for 15 min then for a further 1 h at room temperature. The reaction mixture was then poured onto ice water and extracted into diemthylether. The combined organic phases were washed with brine, dried over MgSO_4_ and concentrated under reduce pressure. The crude material was purified by flash column chromatography: mobile phase 0–50% ethyl acetate gradient in hexanes to give 5-*tert*-Butyl-2-nitrophenol (**8**) (500 mg, 25%). ^1^H NMR (600 MHz, Acetone-*d*_6_) *δ* 10.46 (s, 1H), 8.03 (d, *J* = 9.4 Hz, 1H), 7.23–7.10 (m, 2H), 1.35 (s, 9H); ^13^C NMR (150 MHz, Acetone*-d*_*6*_) *δ* 163.1, 155.4, 132.6, 125.5, 119.0, 117.1, 36.1, 30.9; HRMS (ESI): *m/z* calcd for C_10_H_13_NO_3_ [M—H]^-^ 194.0823 found 194.0814; IR (Neat) ν (cm^-1^): 3233, 2967, 2907, 2871, 1622, 1588, 1530, 1481, 1438, 1369, 1324, 1277, 1228, 1208, 1178. ^1^H NMR data correspond with those reported in the literature [14].

To a solution of **8** (400 mg, 2.05 mmol) in DMF (2 mL) was added excess of K_2_CO_3_ and iodomethane (210 μL, 2.25 mmol). The solution was stirred at room temperature for 16 h. The reaction mixture was diluted with water, extracted with DCM, washed with brine, dried over MgSO_4_ and concentrated under reduce pressure. The crude material was purified by flash column chromatography: mobile phase 0–50% ethyl acetate gradient in hexanes to give **4g** (495 mg, 25%). ^1^H NMR (600 MHz, Acetone-*d*_6_) *δ* 7.78 (d, *J* = 8.5 Hz, 1H), 7.32 (d, *J* = 1.8 Hz, 1H), 7.16 (dd, *J* = 8.5, 1.8 Hz, 1H), 4.00 (s, 3H), 1.37 (s, 9H); ^13^C NMR (150 MHz, Acetone*-d*_*6*_) *δ* 159.4, 153.4, 138.5, 125.7, 118.3, 112.0, 56.8, 36.2, 31.2; IR (Neat) ν (cm^-1^): 2971, 2873, 1604, 1589, 1522, 1493, 1466, 1450, 1406, 1363, 1311, 1287, 1243, 1207, 1190, 1164, 1084, 1026, 917, 857, 843, 826, 698, 656. ^1^H NMR data correspond with those reported in the literature [14].

*N*,*N*-Diethyl-3-methoxy-4-nitroaniline (**4h**). To a solution of 5-fluoro-2-nitroanisle (0.51 g, 3.0 mmol) in DMF (2 mL) was added excess of K_2_CO_3_ and diethylamine (930 μL, 9.0 mmol). The solution was stirred at 90°C for 22 h. The reaction mixture was diluted with acetone, filtered through celite and concentrated under reduced pressure. The crude material was purified by flash column chromatography: mobile phase 0–100% ethyl acetate gradient in hexanes to give **4h** (660 mg, 99%). ^1^H NMR (600 MHz, Acetone-*d*_6_) *δ* 7.92 (d, *J* = 9.4 Hz, 1H), 6.35 (dd, *J* = 9.4, 2.4 Hz, 1H), 6.29 (d, *J* = 2.4 Hz, 1H), 3.94 (s, 3H), 3.53 (q, *J* = 7.1 Hz, 4H), 1.22 (t, *J* = 7.1 Hz, 6H); ^13^C NMR (150 MHz, Acetone*-d*_*6*_) *δ* 156.8, 153.1, 128.6, 127.4, 103.1, 94.2, 55.5, 44.5, 11.8; IR (Neat) ν (cm^-1^): 2974, 2933, 1602, 1571, 1515, 1483, 1449, 1403, 1379, 1341, 1313, 1263, 1230, 1194, 1097, 1078, 1019, 811, 749, 698. ^1^H NMR data correspond with those reported in the literature [15].

2-Methoxy-1-nitronaphthalene (**4i**). The methylation was performed according to the procedure reported by Kumar *et al* [16]. To a solution of 1-nitro-2-naphthol (380 mg, 2.0 mmol) in acetone (5 mL) was added KOH (340 mg, 6.0 mmol) and iodomethane (370 μL, 4.0 mmol). The solution was refluxed overnight. The reaction mixture was cooled to room temperature. Water was then added to the reaction mixture and extracted with DCM. The combined organic layer was dried over Na_2_SO_4_ and concentrated under reduced pressure. The crude material was purified by flash column chromatography: mobile phase 0–100% ethyl acetate gradient in hexanes to give **4i** (350 mg, 87%). ^1^H NMR (600 MHz, Acetone-*d*_6_) *δ* 8.17 (d, *J* = 9.2 Hz, 1H), 8.01 (d, *J* = 8.3 Hz, 1H), 7.72–7.58 (m, 3H), 7.53 (ddd, *J* = 8.0, 6.9, 1.0 Hz, 1H), 4.09 (s, 3H); ^13^C NMR (150 MHz, Acetone*-d*_*6*_) *δ* 149.5, 136.8, 133.2, 130.1, 129.2, 129.1, 126.2, 126.0, 120.6, 114.7, 57.6; HRMS (ESI): *m/z* calcd for C_11_H_9_NO_3_ [M + H]^+^ 204.0655 found 204.0654; IR (Neat) ν (cm^-1^): 1636, 1559, 1522, 1474, 1457, 1436, 1355, 1282, 1155, 1079, 866, 809, 795, 777, 750, 669, 650. ^1^H NMR data correspond with those reported in the literature [16].

### General experimental procedure for the synthesis of *tert*-butoxy nitrobenzenes (5a-5i)

A mixture of nitrobenzene (0.3 mmol), sodium *tert*-butoxide (7.0 equiv.), in dry toluene (1.5 mL) or in dry toluene/dry DME (9:1) was reacted in a microwave reactor. The reaction mixture was diluted with DCM, filtered through Celite, and concentrated under reduce pressure. The crude material was purified by flash column chromatography (mobile phase: 0–100% ethyl acetate gradient in hexanes) to give the product.

#### Spectroscopic data of products

1-*tert*-Butoxy-2-nitrobenzene (**5a**). ^1^H NMR (600 MHz, CDCl_3_) *δ* 7.71 (dd, *J* = 8.1, 1.7 Hz, 1H), 7.46 (ddd, *J* = 8.3, 7.5, 1.7 Hz, 1H), 7.22 (dd, *J* = 8.3, 1.2 Hz, 1H), 7.17–7.07 (m, 1H), 1.42 (s, 9H); ^13^C NMR (150 MHz, CDCl_3_) *δ* 149.2, 145.6, 132.7, 124.9, 124.6, 122.8, 82.8, 28.9; HRMS (ESI): m/z calcd for C_10_H_14_NO_3_ [M + H]^+^, 196.0968, found 196.0963; IR (Neat) ν (cm^-1^): 2982, 1603, 1527, 1481, 1368, 1266, 1244, 1156, 900, 846, 769, 669, 659.

1-*tert*-Butoxy-4-nitrobenzene (**5b**). ^1^H NMR (600 MHz, CDCl_3_) *δ* 8.16 (d, *J* = 9.2 Hz, 2H), 7.05 (d, *J* = 9.2 Hz, 2H), 1.46 (s, 9H); ^13^C NMR (150 MHz, CDCl_3_) *δ* 161.9, 142.5, 125.2, 121.7, 80.6, 28.9; HRMS (ESI): m/z calcd for C_10_H_12_NO_2_ [M—H]^-^, 194.0823, found 194.0812; IR (Neat) ν (cm^-1^): 2980, 1604, 1590, 1515, 1492, 1370, 1343, 1263, 1163, 1112, 897.

1-*tert*-Butoxy-4-methoxy-2-nitrobenzene (**5c**). ^1^H NMR (600 MHz, CDCl3) *δ* 7.24 (d, *J* = 3.1 Hz, 1H), 7.12 (d, *J* = 9.1 Hz, 1H), 7.02 (dd, *J* = 9.1, 3.2 Hz, 1H), 3.82 (s, 3H), 1.35 (s, 9H); ^13^C NMR (150 MHz, CDCl_3_) *δ* 154.9, 145.8, 142.4, 126.6, 119.5, 108.9, 82.4, 55.9, 28.6; HRMS (ESI): m/z calcd for C_11_H_15_NO_4_Na [M + Na]^+^, 248.0893, found 248.0890; IR (Neat) ν (cm^-1^): 2979, 2937, 1571, 1534, 1496, 1463, 1442, 1392, 1367, 1308, 1290, 1272, 1222, 1160, 1037, 878, 836, 813, 794.

2-*tert*-Butoxy-4-fluoro-1-nitrobenzene (**5d**). ^1^H NMR (600 MHz, CD_3_CN) *δ* 7.80 (dd, *J* = 9.1, 6.1 Hz, 1H), 7.08 (dd, *J* = 10.5, 2.6 Hz, 1H), 6.94 (ddd, *J* = 9.1, 7.8, 2.6 Hz, 1H), 1.41 (s, 9H); ^13^C NMR (150 MHz, CD_3_CN) *δ* 165.3 (d, *J* = 252.6 Hz), 151.9 (d, *J* = 11.9 Hz), 127.6 (d, *J* = 11.5 Hz), 112.1 (d, *J* = 24.6 Hz), 110.8 (d, *J* = 23.9 Hz), 84.7, 28.7; HRMS (ESI): m/z calcd for C_10_H_12_FNO_3_ [M—*t*Bu]^-^ 156.0102 found 156.0095; IR (Neat) ν (cm^-1^): 2984, 1616, 1588, 1558, 1528, 1480, 1370, 1355, 1281, 1175, 1147, 1088, 988, 843, 669. ^1^H NMR data correspond with those reported in the literature [6]_._

4-Bromo-2-*tert*-butoxy-1-nitrobenzene (**5e**). ^1^H NMR (600 MHz, CDCl_3_) *δ* 7.62 (d, *J* = 8.6 Hz, 1H), 7.38 (d, *J* = 2.0 Hz, 1H), 7.26 (dd, *J* = 8.6, 2.0 Hz, 1H), 1.44 (s, 9H); ^13^C NMR (150 MHz, CD_3_CN) *δ* 150.5, 145.5, 128.3, 127.1, 127.0, 126.9, 84.9, 28.9; HRMS (ESI): m/z calcd for C_10_H_12_BrNO_3_ [M—*t*Bu]^-^ 215.9302 found 215.9295; IR (Neat) ν (cm^-1^): 2981, 2936, 1595, 1561, 1527, 1473, 1397, 1369, 1290, 1261, 1157, 1097, 1071, 932, 914, 882, 844, 819, 763. ^1^H NMR data correspond with those reported in the literature [17].

1-Bromo-5-*tert*-butoxy-2-methoxy-4-nitrobenzene (**5f**). ^1^H NMR (600 MHz, CD_3_CN) *δ* 7.50 (s, 1H), 7.37 (s, 1H), 3.89 (s, 3H), 1.32 (s, 9H); ^13^C NMR (150 MHz, CD_3_CN) *δ* 152.7, 145.9, 142.9, 130.8, 116.4, 108.4, 84.0, 57.8, 28.8; HRMS (ESI): m/z calcd for C_7_H_5_BrNO_4_ [M—*t*Bu]^-^ 245.9407 found 245.9406; IR (Neat) ν (cm^-1^): 2979, 2938, 1560, 1526, 1485, 1440, 1392, 1368, 1310, 1264, 1218, 1156, 1042, 966, 894, 873, 825, 795, 762.

2-*tert*-Butoxy-4-tert-butyl-1-nitrobenzene (**5g**). ^1^H NMR (600 MHz, CD_3_CN) *δ* 7.68 (d, *J* = 8.5 Hz, 1H), 7.28–7.22 (m, 2H), 1.36 (s, 9H), 1.32 (s, 9H); ^13^C NMR (150 MHz, CD_3_CN) *δ* 158.4, 149.4, 144.1, 125.2, 123.2, 121.3, 83.3, 35.8, 31.1, 29.0; HRMS (ESI): m/z calcd for C_10_H_12_NO_3_ [M—*t*Bu]^-^ 194.0823 found 194.0817; IR (Neat) ν (cm^-1^): 2970, 1602, 1584, 1522, 1487, 1459, 1404, 1367, 1355, 1280, 1233, 1160, 959, 843.

3-*tert*-Butoxy-*N*,*N-*diethyl-4-nitroaniline (**5h**). ^1^H NMR (600 MHz, Acetone*-d*_*6*_) *δ* 7.85 (d, *J* = 9.0 Hz, 1H), 6.49–6.51 (m, 1H), 6.37 (d, *J* = 3 Hz, 1H), 3.47–3.51 (m, 4H), 1.40 (s, 9H), 1.21 (t, *J* = 7.2 Hz, 6H); ^13^C NMR (150 MHz, Acetone*-d*_*6*_) *δ* 153.4, 152.8, 133.8, 128.7, 106.9, 106.6, 82.5, 45.3, 29.0, 12.7; HRMS (ESI): m/z calcd for C_14_H_23_N_2_O_3_ [M + H]^+^, 267.1703, found 267.1703; IR (Neat) ν (cm^-1^): 2973, 2928, 1601, 1561, 1519, 1488, 1449, 1435, 1405, 1388, 1376, 1364, 1350, 1319, 1297, 1283, 1258, 1218, 1198, 1164, 1154, 1096, 1074, 885, 859, 837, 819, 799, 757, 747, 717, 696, 669, 652.

2-*tert*-Butoxy-1-nitronaphthalene (**5i**). ^1^H NMR (600 MHz, CDCl_3_) *δ* 7.85 (t, *J* = 9.1 Hz, 2H), 7.66 (d, *J* = 8.5 Hz, 1H), 7.58 (ddd, *J* = 8.4, 6.9, 1.1 Hz, 1H), 7.48 (ddd, *J* = 8.1, 7.0, 1.0 Hz, 1H), 7.38 (d, *J* = 9.1 Hz, 1H), 1.47 (s, 9H). ^13^C NMR (150 MHz, CDCl3) *δ* 145.8, 141.8, 130.8, 129.5, 128.6, 127.9, 125.8, 125.6, 122.1, 120.9, 82.8, 29.3; HRMS (ESI): m/z calcd for C_10_H_6_NO_3_ [M—*t*Bu]^-^ 188.0353 found 188.0346; IR (Neat) ν (cm^-1^): 2975, 2929, 2870, 1600, 1563, 1519, 1488, 1449, 1435, 1405, 1389, 1376, 1365, 1350, 1298, 1259, 1219, 1199, 1164, 1094, 1073, 886, 859, 838, 820, 799, 756, 747, 718, 697, 610.

2-Ethoxy-4-methoxy-1-nitrobenzene (**6**). A mixture of 2,4-dimethoxy-1-nitrobenzene (13 mg, 0.076 mmol) and sodium ethoxide (21 wt % solution, 199 μL, 0.532 mmol) in toluene (2 mL) was stirred for 20 hours at room temperature. The mixture was diluted with EtOAc, washed by water and brine, dried over Na_2_SO_4_ anhydrous, and filtered. The filtrate was concentrated using rotavapor, purified by flash column chromatography to afford 2-ethoxy-4-methoxy-1-nitrobenzene (14.8 mg, 0,075 mmol, 99%). ^1^H NMR (600MHz, CDCl_3_) *δ* 7.96 (d, *J* = 9.0 Hz, 1H), 6.56–6.44 (m, 2H), 4.15 (q, *J* = 7.0 Hz, 2H), 3.87 (s, 3H), 1.49 (t, *J* = 7.0 Hz, 3H); ^13^C NMR (150 MHz, CDCl_3_) *δ* 164.7, 155.1, 133.3, 128.3, 104.8, 100.5, 65.4, 56.0, 14.6; HRMS (ESI): *m/z* calcd for C_11_H_9_NO_4_ [M + H]^+^ 198.0761 found 198.0758; IR (Neat) ν (cm^-1^): 3128, 3074, 2992, 2952, 2845, 1621, 1590, 1514, 1498, 1471, 1451, 1394, 1370, 1341, 1277, 1227, 1185, 1152, 1110, 1090, 1037, 985, 889, 846, 824, 752, 728, 689, 653, 640, 587, 569. ^1^H NMR data correspond with those reported in the literature [18].

2-(Cyclohexyloxy)-4-methoxy-1-nitrobenzene (**7**). A mixture of sodium metal (460 mg, 20 mmol) and cyclohexanol (2.113 mL, 20 mmol) in dry THF (25mL) was refluxed until sodium metal thoroughly disappeared. The reaction mixture was concentrated under reduced pressure. The crude sodium salt of cyclohexanol was used without further purification.

A mixture of 2,4-dimethoxy-1-nitrobenzene (55 mg, 0.3 mmol) and sodium salt of cyclohexanol (256 mg, 2.1 mmol) in toluene (1.5 mL) was stirred for 24 hours at room temperature. The reaction mixture was diluted by DCM, filtered through celite, and concentrated under reduced pressure. The crude mixture was purified by flash chromatography to give **7** (57 mg, 0.226 mmol, 75%). ^1^H NMR (600 MHz, CDCl_3_) *δ* 7.93 (d, *J* = 9.1 Hz, 1H), 6.53 (d, *J* = 2.5 Hz, 1H), 6.48 (dd, *J* = 9.1, 2.5 Hz, 1H), 4.41 (tt, *J* = 7.7, 3.4 Hz, 1H), 3.86 (s, 3H), 1.97–1.89 (m, 2H), 1.88–1.76 (m, 2H), 1.76–1.65 (m, 2H), 1.57–1.48 (m, 1H), 1.45–1.34 (m, 3H); ^13^C NMR (150 MHz, CD_3_CN) *δ* 165.5, 154.5, 135.0, 128.7, 106.5, 102.6, 77.8, 56.8, 31.9, 26.2, 23.8; HRMS (ESI): *m/z* calcd for C_13_H_17_NO_4_ [M + H]^+^ 252.1230 found 252.1224; IR (Neat) ν (cm^-1^): 2937, 2859, 1607, 1581, 1513, 1447, 1345, 1315, 1290, 1208, 1172, 1097, 1035, 1018, 985, 842.

### Determination of conversion yield using HPLC

#### Generation of external standard calibration curves

Stock solutions of standard compounds **1**, **2**, and **3** with concentrations between 0.6 mM and 0.009375 mM in Acetonitrile/water/formic acid (20: 80: 0.1) were prepared and analyzed by HPLC. The external standard calibration curve of each compound was generated by plotting the data as AUC concentration (mM).

#### Determining conversion yield using external standard calibration curves

After reaction, the reaction mixture was diluted by dichloromethane and filtered through celite. The filtrate was concentrated under reduced pressure and redissolved in 5.0 mL of acetonitrile. The solution was 100-fold diluted in acetonitrile/water/formic acid (20: 80: 0.1) and the mixture was analyzed by HPLC. Using this data and the external standard calibration curves, the yield of **1**, **2**, and **3** in the reaction mixture was determined.

## Results and discussion

**Fig 2:**
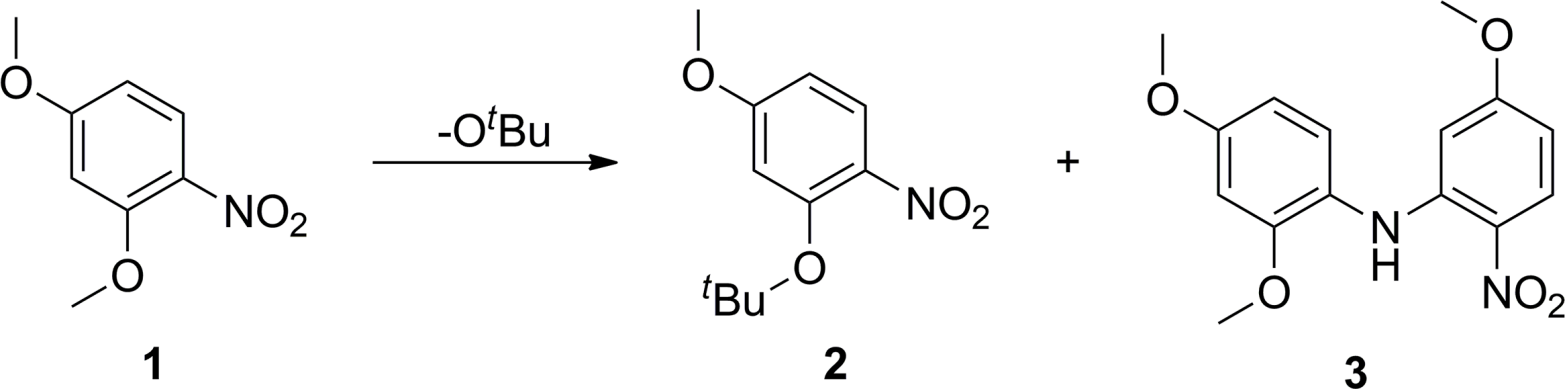
S_N_Ar reaction of 2,4-dimethoxynitrobenzene with *tert*-butoxide.

**Fig 3:**
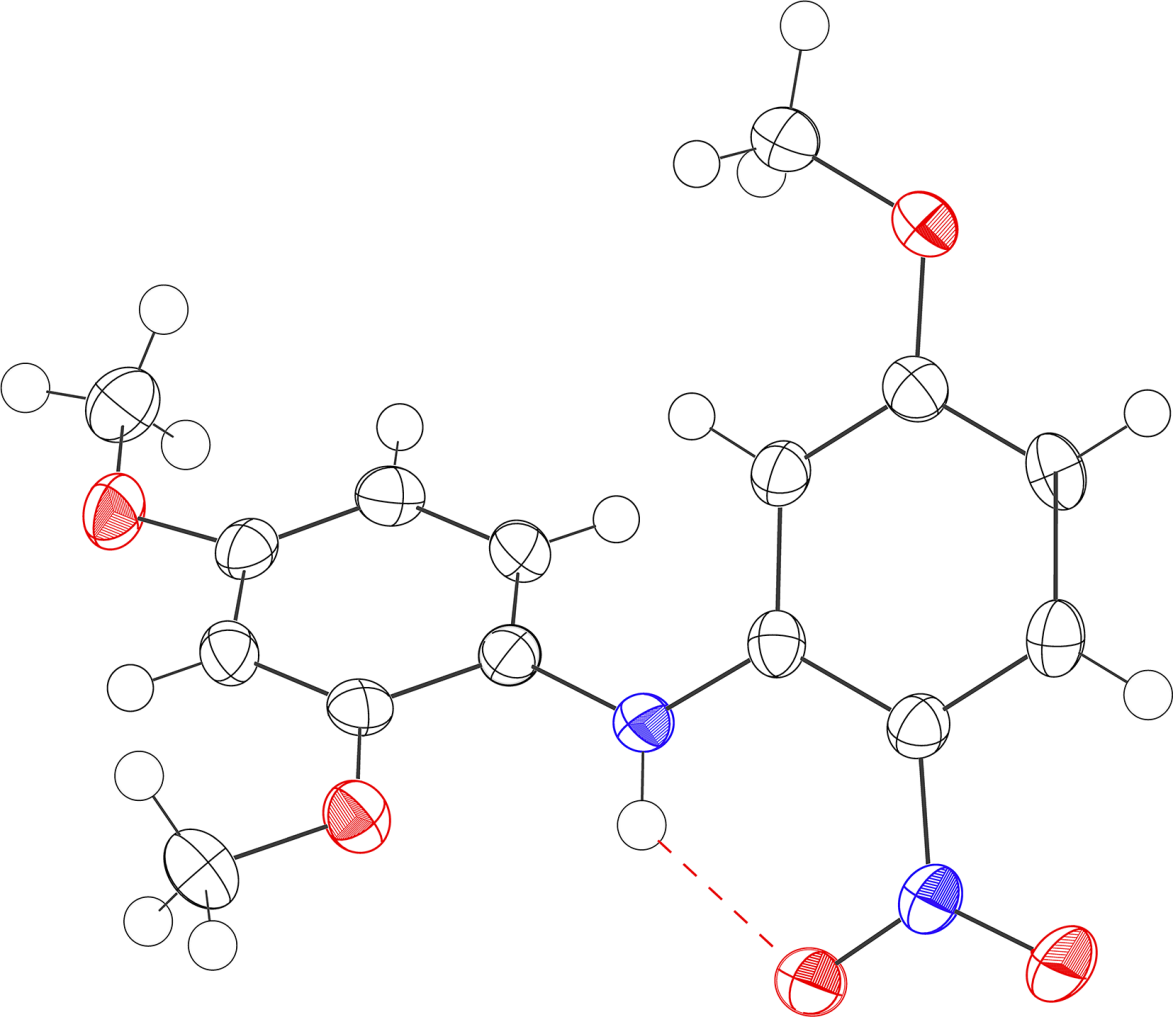
X-ray crystal structure of 3.

The S_N_Ar reactions were carried out on 2,4-dimethoxynitrobenzene in a microwave reactor at various reaction temperatures with different time points to determine the ortho selectivity and yield of the reaction (Fig 2, Table 1). Yields were estimated by analyzing the quantity of each product in the crude reaction mixture using HPLC, as determined by standard curves for each compound. Treatment of 2,4-dimethoxynitrobenzene (**1**) with sodium tert-butoxide for 10 min at 100°C provided ortho-*t*-butoxynitrobenzene **2** in 13% yield (Table 1, entry 1). By running the reaction for longer time, the yield of **2** was improved up to 40% (Table 2, entries 2–4). However, the starting material disappeared completely within 40 min, but a large amount of byproducts were formed (Table 1, entry 4). Although *ortho*-*t*-butoxy compound **2** was formed in higher yield as the temperature increased, the exposure of the reaction mixture to excessive heating or extended reaction times led to the formation of byproducts including 2-nitroaniline derivative **3**, which was obtained as a major byproduct at over 130°C (Table 1, entries 10–13). We expected that the major byproduct might be an azoxy compound since nitrobenzene can be reduced in the presence of sodium hydroxide or sodium methoxide [19, 20]. Unexpectedly, X-ray crystallography revealed that the byproduct was 2-nitroaniline **3** (Fig 3), which crystallized from acetone [21]. The *in situ* generated aniline species is expected to attack 2,4-dimethoxy nitrobenzene at the *ortho* position to form compound **3** by virtue of an S_N_Ar reaction. Interestingly, *para*-*t*-butoxy compounds were not detectable under any conditions, indicating that the *ortho* substitution is highly dominant over the *para* substitution in this reaction.

Next, the amount of nucleophile NaO^*t*^Bu was gradually increased from 1 to 10 equiv. (Fig 2, Table 2), which enhanced product formation within a limited reaction time. Noticeably, the formation of product **2** was observed to reach a maximum yield of 72% without any detectable byproducts using 7 equiv. of NaO^*t*^Bu at 110°C for 20 min (Table 2, entry 9). Byproducts including **3** were detected when the reaction was performed using 7 equiv. of NaO^*t*^Bu for 30 min or 10 equiv. of NaO^*t*^Bu for 20 min (Table 2, entries 10 and 11).

The effect of the counterion of *t*-butoxide on the S_N_Ar reaction was investigated (Table 2, entries 7–20). The use of lithium *t*-butoxide did not afford the desired product **2**, but resulted in substrate decomposition at all temperatures, leading to the formation of byproduct **3** at 160°C. In the presence of potassium *t*-butoxide, a complete loss of substrate was observed without the formation of the desired product **2** at 90 and 110°C. Only at 60°C, KO^*t*^Bu afforded the product in about 40% yield. Overall, the sodium ion can be considered as the best counterion for *t*-butoxide in these S_N_Ar reactions.

The reactions were carried out at several different concentrations to determine the optimal reaction concentration (Fig 2, Table 3). Reactions at 0.05 and 0.1 M resulted in moderate conversions (Table 3, entries 1 and 2). A low conversion was also observed even at high concentrations such as 0.5 M (Table 3, entry 4), which was expected since the reaction mixture turned into a sticky semi-solid and lost homogeneity. As shown in Table 3, the highest conversion was observed at 0.2 M without the formation of the determined byproduct **3**. Therefore, a reaction concentration of 0.2 M was employed for further optimization of the S_N_Ar reaction.

To explore the effect of the solvent on the *o*-selectivity and conversion, a series of non-polar, polar-aprotic, and polar-protic solvents were screened (Fig 2, Table 4). As shown in Table 4, toluene was the most effective solvent in this reaction among the conditions when using only a single solvent. The use of xylene afforded adduct **2** in good yield but was less effective than toluene. It has been reported that toluene was the best solvent to attain high *ortho* selectivity in S_N_Ar reactions with 2,4-dihalonitrobenzene [6] and a six-membered transition state generated by a metal alkoxide, the nitro group of the substrate, and the nucleophile was proposed to be stabilized by non-polar solvents such as toluene, while polar solvents reduced the *ortho*-selectivity rather than preventing the progress of the reaction [22]. However, in the S_N_Ar reactions of 2,4-dimethoxynitrobenzene, polar solvents, with the exception of HMPA, hampered the reactions, leading to severe decomposition of the substrate. Interestingly, we found that the use of 10% of dimethoxyethane (DME) as a co-solvent with toluene increased the conversion to the desired compound **1** without producing byproducts including **3**, compared to toluene alone. It could be surmised that a small amount of DME may positively be involved in suppressing the reduction of the substrate to an anilino-species to form **3** or the substrate decomposition through stabilization of the Meisenheimer complex at high temperatures (110°C). However, the use of large amounts of DME gave negative results similarly to polar solvents. The use of 10% of diethyl ether also gave a substantially lower conversion.

**Fig 4:**
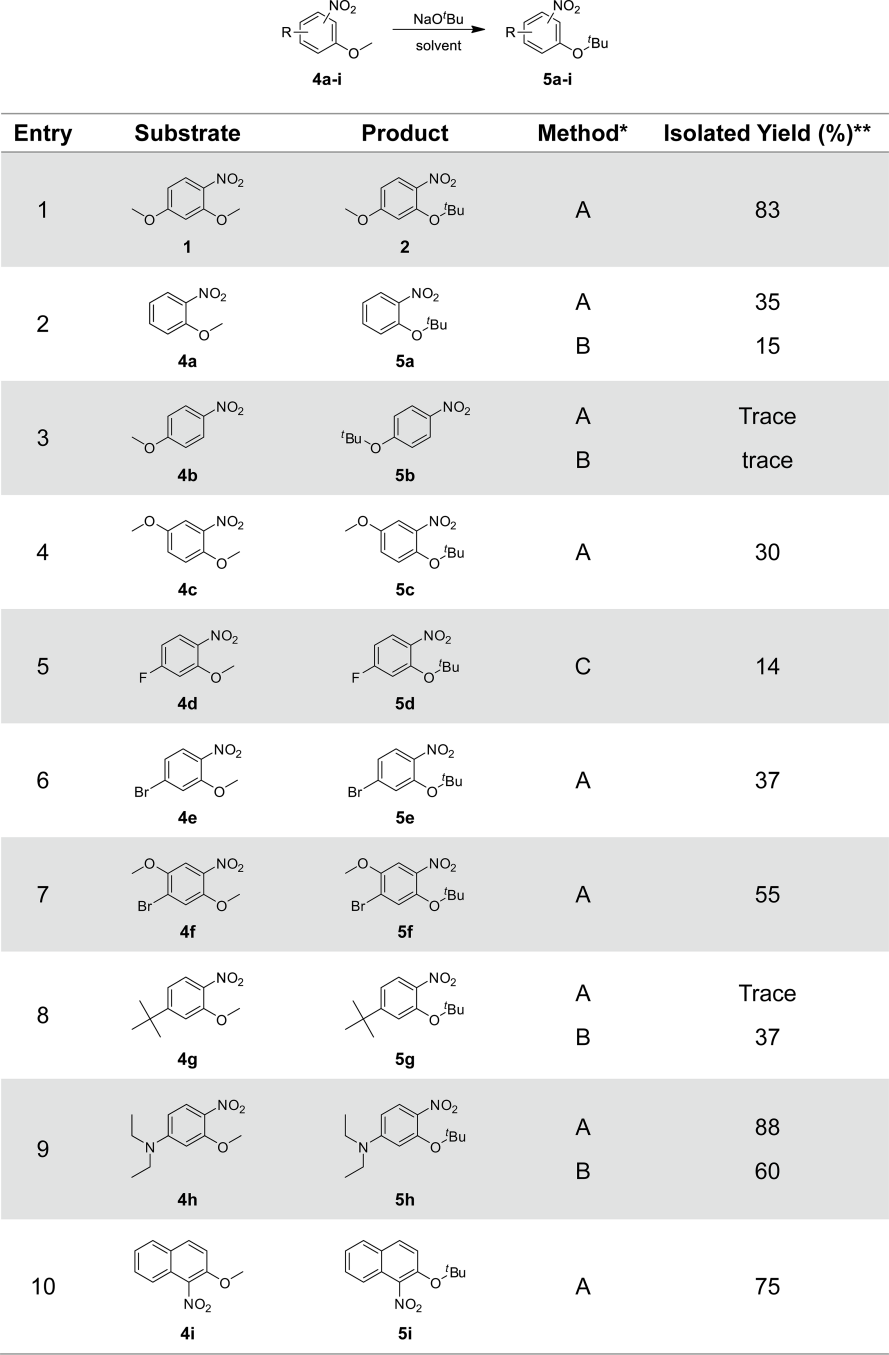
Transetherification of *o*-methoxynitrobenzenes with sodium *tert*-butoxide. *Methods: A. nitrobenzene derivative (0.3 mmol) and NaO^*t*^Bu (2.1 mmol) in 10% DME in toluene (1.5 mL) at 110°C for 20 min under microwave irradiation. B. nitrobenzene derivative (0.3 mmol) and NaO^*t*^Bu (2.1 mmol) in toluene (1.5 mL) at 110°C for 20 min under microwave irradiation. C. nitrobenzene derivative (0.3 mmol) and NaO^*t*^Bu (0.6 mmol) in toluene (1.5 mL) at room temperature for 5 d. ** Isolated yield after purification.

Finally, the optimized reaction conditions for the S_N_Ar reaction of 2,4-dimethoxynitrobenzene with sodium *t*-butoxide were applied to various *ortho*-methoxynitrobenzenes (Fig 4). Each conversion was determined by measuring the corresponding isolated yield. The reaction of substrate **1** with NaO*t*Bu gave product **2** in 83% isolated yield, which was similar to the yield (87%) calculated by HPLC analysis with a standard curve (Table 2, entry 13; Fig 4, entry 1). The reaction of 2-methoxynitrobenzene **4a** afforded *t*-butoxy compound **5a** in only 35% yield, less than half that of 2,4-dimethoxynitrobenzene **1** (Fig 4, entry 2). 4-methoxynitrobenzene **4b** was poorly substituted by *t*-butoxide (Fig 4, entry 3). In this type of S_N_Ar reaction where a methoxy group acts as a leaving group, it seems that the *ortho* substitution largely dominates over the *para* substitution, compared to the case of dihalonitrobenzenes. This suggests a significant difference of reactivity between the *ortho* and *para* positions, although the *ortho* selectivity might be caused by chelation effects directing the nucleophile. An explanation for the observation of the higher conversion of dimethoxy compound **1** compared to that of *ortho*-methoxy compound **4a** could be that the para-methoxy group may act positively in forming a complex of sodium cation, nitrobenzene and nucleophile through resonance effect. For compound **4c** with a *meta*-methoxy group, the resonance effect was obviously disadvantageous compared to **1** (Fig 4, entry 4). Interestingly, the 2-*t*-butoxy-4-fluoro product **5d** was obtained from **4d** at room temperature, although in only 14% yield (Fig 4, entry 5). A chelation effect may form a little amount of *ortho*-adduct **5d**, instead of the substitution of *t*-butoxide for the most reactive fluoro group. Bromo compounds **5e** and **5f** were formed in higher yields, i.e. 37% and 55% yield, respectively, than fluoro compound **5d** at higher temperatures (Fig 4, entries 6 and 7). *Para*-*t*-butyl compound **4g** was not converted to **5g** under the optimized conditions (method A, Fig 4, entry 8). However, the reaction of **4g** under DME-free conditions (method B) gave **5g** in about 30% yield (Fig 4, entry 8). This suggests that the effect of DME may differ depending on the substrate. 4-Diethylamino compound **4h** and naphtyl compound **4i** were converted to the corresponding *t*-butoxy compounds **5h** and **5i** in good yields, respectively (Fig 4, entries 9 and 10).

**Fig 5:**
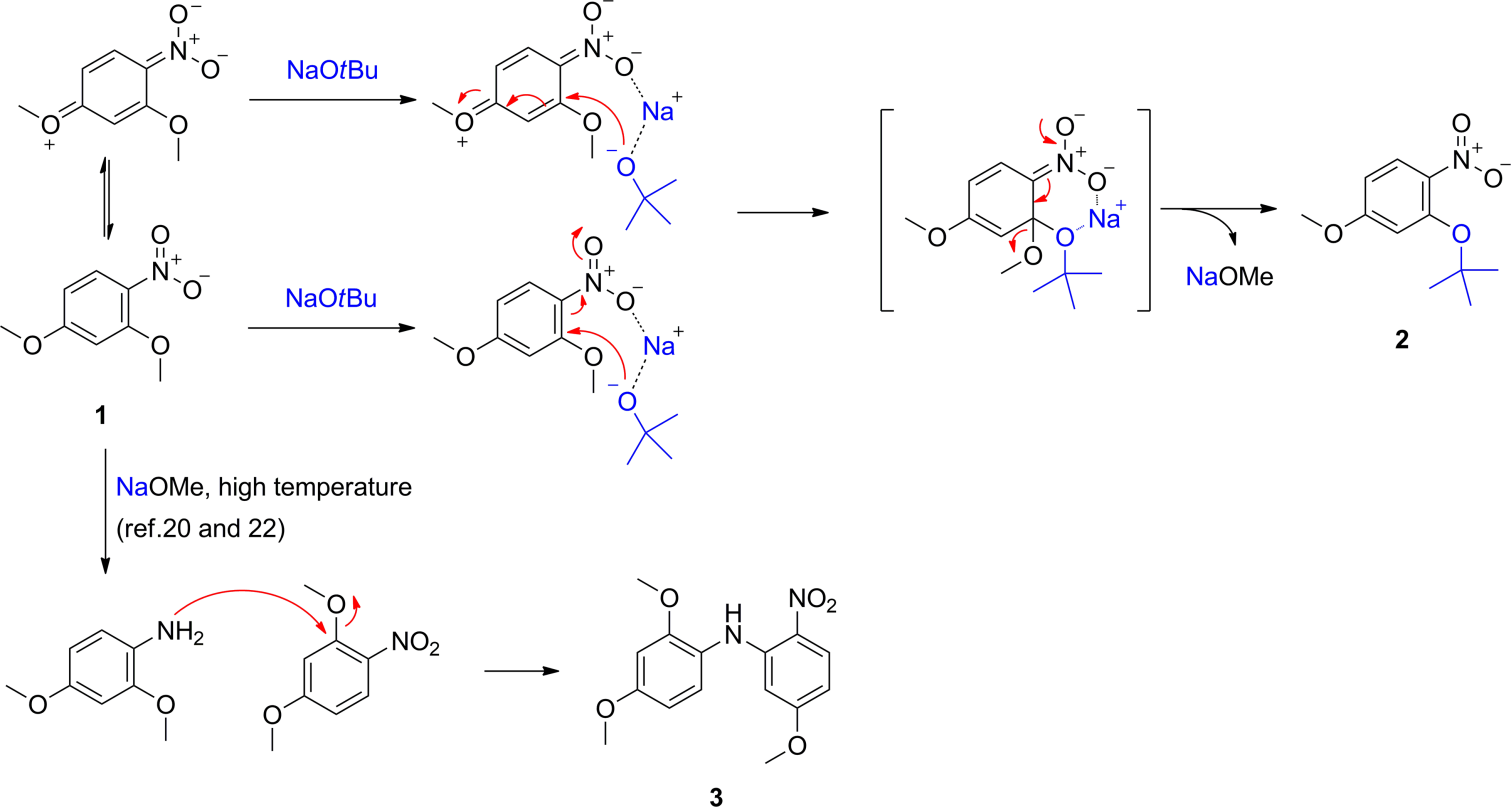
Proposed mechanism for the formation of 2 and 3.

Fig 5 exhibits possible mechanistic pathways for transetherification, which could account for *ortho*-selectivity and high conversion of *para*-methoxy and *para*-diethylamino substrates to **2**. With regard to the formation of byproduct **3**, nitrobenzene could be reduced to aniline in the presence of alcohol and base [23]. A small amount of nitrobenzene may be reduced to aniline in the presence of methoxide released from substrate at high temperature, which may quickly react with substrate **1** to give byproduct **3**. However, further investigation is required to clarify the mechanisms.

**Fig 6:**
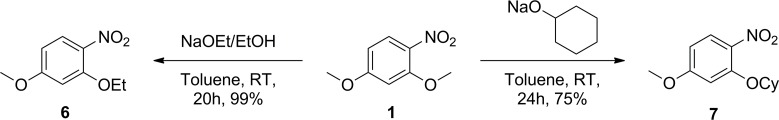
S_N_Ar reactions of sodium ethoxide and sodium cyclohexanolate.

As application of this transetherification, the use of sodium ethoxide and sodium cyclohexanolate as nucleophiles afforded ethoxy and cyclohexyloxy products in 99% and 75% of the yield, respectively (Fig 6). Reaction of sodium ethoxide with **1** in toluene at room temperature for 20 h gave **6** in excellent yield. Conversion of **1** to cyclohexyloxy product **7** was also accomplished at room temperature. Thus, various nucleophiles might be introduced at the *ortho* position by S_N_Ar reactions under a controlled condition.

**Fig 7:**
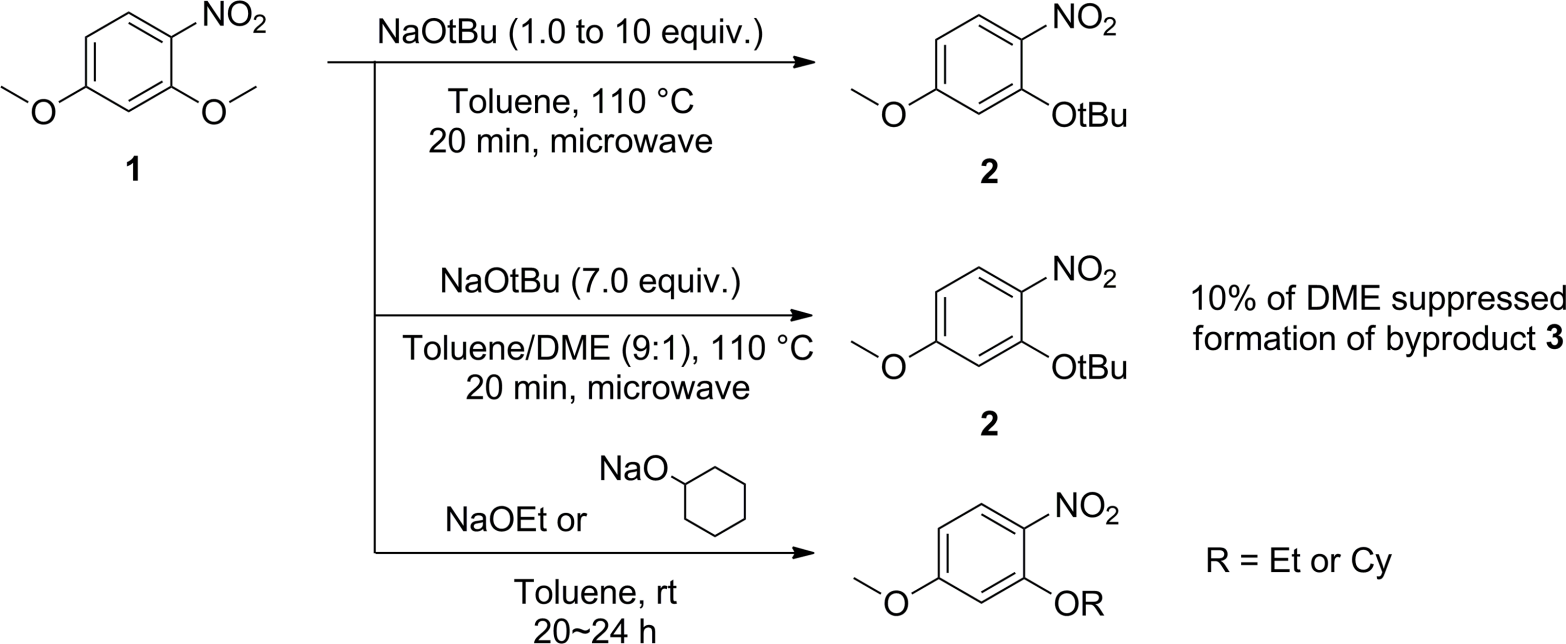
Summary of reaction conditions.

In conclusion, the S_N_Ar reaction of 2,4-dimethoxynitrobenzene with bulky *t*-butoxide was investigated under various conditions, which are summarized in Fig 7. The outcome of the reaction was affected by temperature, time, concentration, solvent, metal alkoxide, and type of substrate. *Ortho* selectivity in the S_N_Ar reaction of 2,4-dimethoxynitrobenzene was observed to be as excellent as that in 2,4-dihalonitroarenes like 2,4-difluoronitrobenzene, although this reaction needed high temperature and limited reaction time under microwave irradiation. The highest conversion of 2,4-dimethoxynitrobenzene to the corresponding *ortho*-*t*-butoxide compound was observed in toluene with 10% of DME as the co-solvent. Anilinonitrobenzene derivative **3** was identified as a major byproduct of the reaction in polar solvents or at high temperatures. It was suggested that a small amount of DME might suppress the formation of byproducts in the S_N_Ar of 2,4-dimethoxynitrobenzene. S_N_Ar reactions of methoxy groups of nitrobenzenes needed specific conditions for each substrate, in contrast to those of halogen groups of activated aromatics. Although this S_N_Ar reaction is very sensitive to small changes in the reaction conditions, the S_N_Ar reactions of 2,4-dimethoxynitrobenzene with *t*-butoxide proceeded in good yields. This study could help in understanding the S_N_Ar reaction of methoxy groups of activated aromatics.

## Supporting information

S1 File Contains ^1^H and ^13^C NMR spectra of all compounds and crystallographic data of byproduct 3. (PDF)
