# Supplementary material for: Transetherification of 2,4-dimethoxynitrobenzene by aromatic nucleophilic substitution
Source: PLoS One. 2017 Aug 23;12(8):e0183575. doi: 10.1371/journal.pone.0183575 (PMC5568332; doi:10.1371/journal.pone.0183575)
Supplement: S1 File — (PDF) [file pone.0183575.s001.pdf]

Supporting information

Transetherification of 2,4-Dimethoxynitrobenzene by Aromatic Nucleophilic Substitution

Jiho Song[1], Hae Ju Kang[1], Jung Wuk Lee[1], Michelle A. Wenas[1], Seung Hwarn Jeong[1], Taeho Lee[2], Kyungsoo Oh[1], Kyung Hoon Min[1,*]

[1]College of Pharmacy, Chung-Ang University, Seoul 06974, Republic of Korea

[2]College of Pharmacy, Kyungpook National University, Daegu 41566, Republic of Korea

E-mail: khmin@cau.ac.kr (KHM)

1. ^{1}H and ^{13}C NMR spectra

^{1}H NMR (600 MHz, CD_3CN) and ^{13}C NMR (150 MHz, CD_3CN) spectra of compound **2**

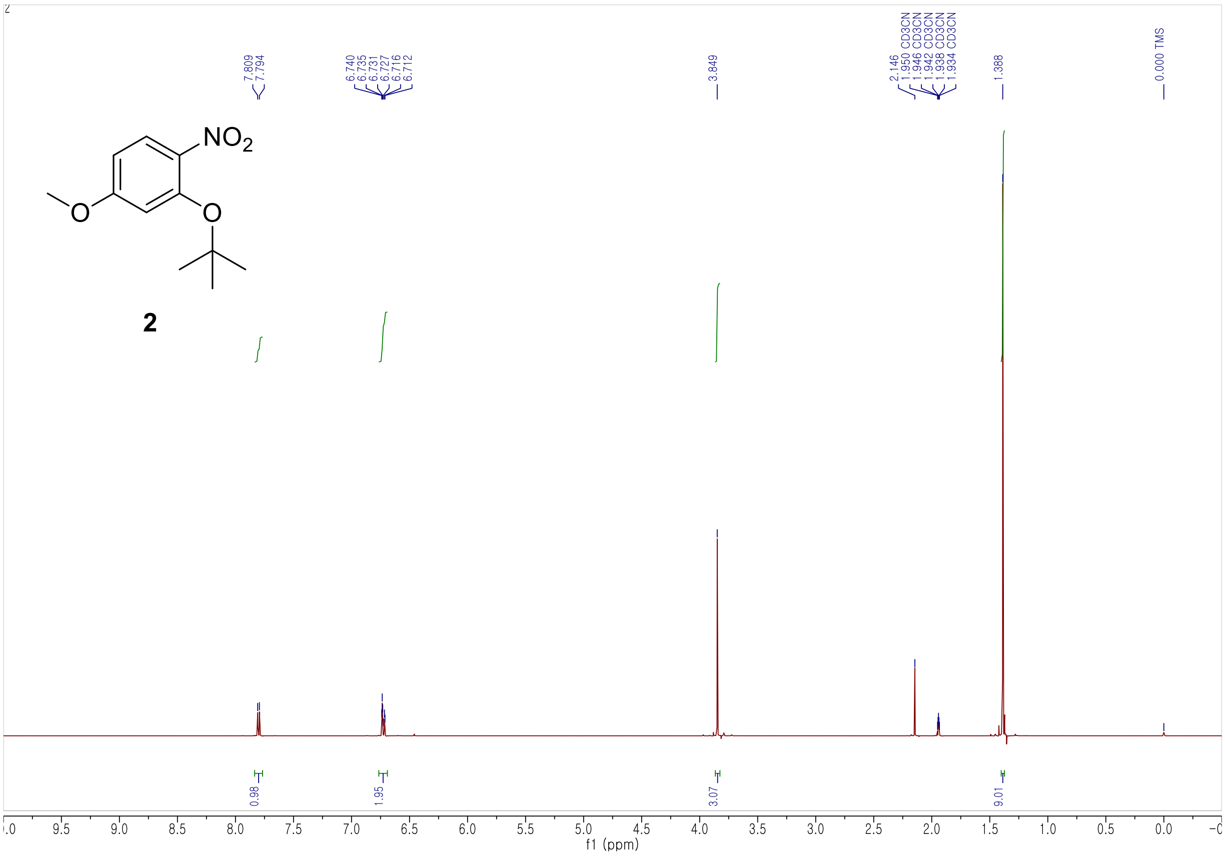

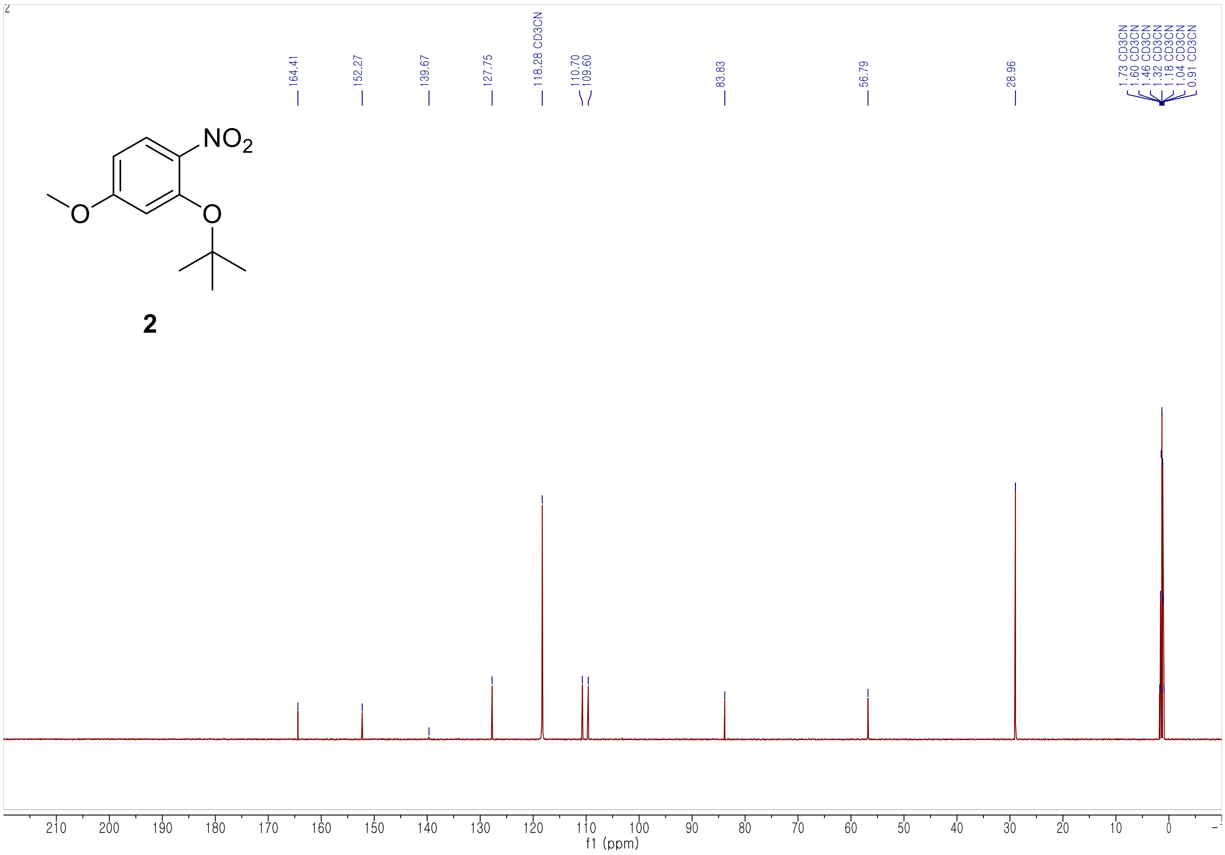

^{1}H NMR (600 MHz, CD_3CN) and ^{13}C NMR (150 MHz, CD_3CN) spectra of compound **3**

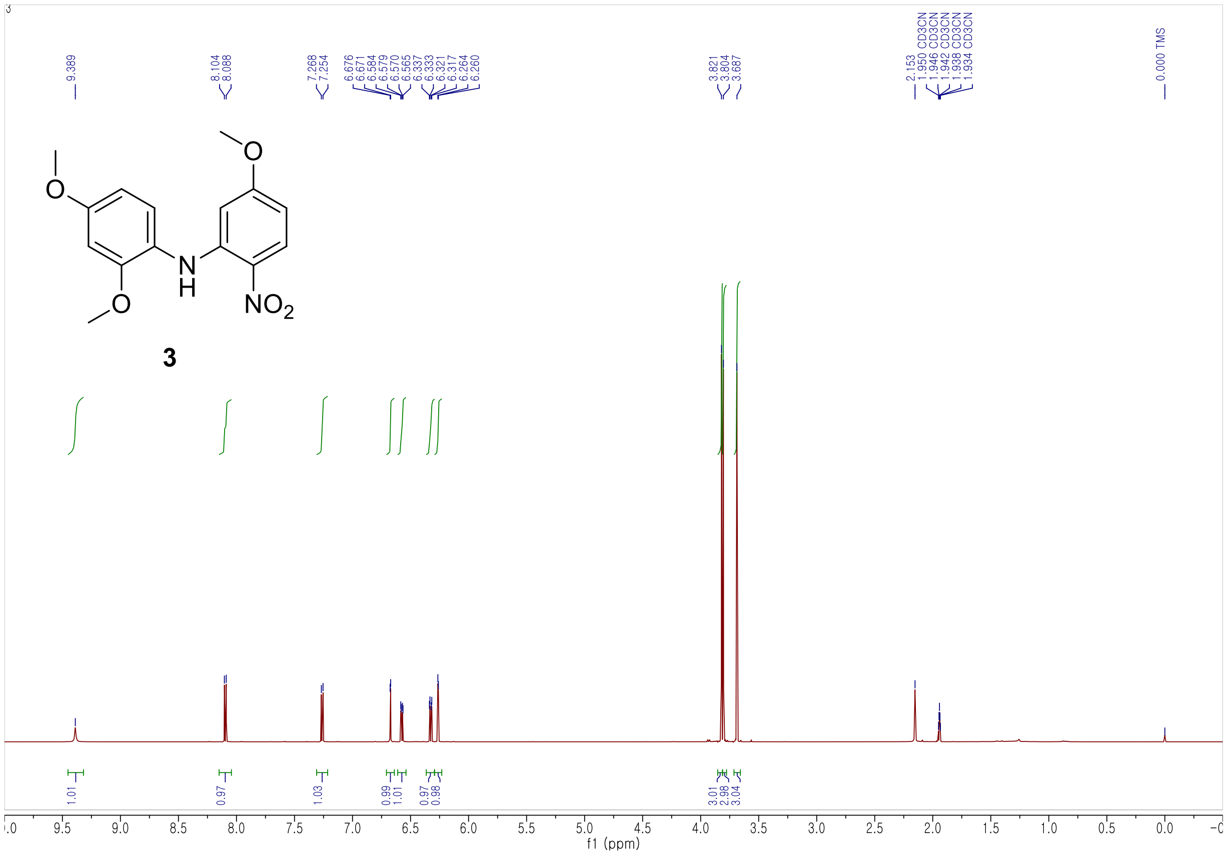

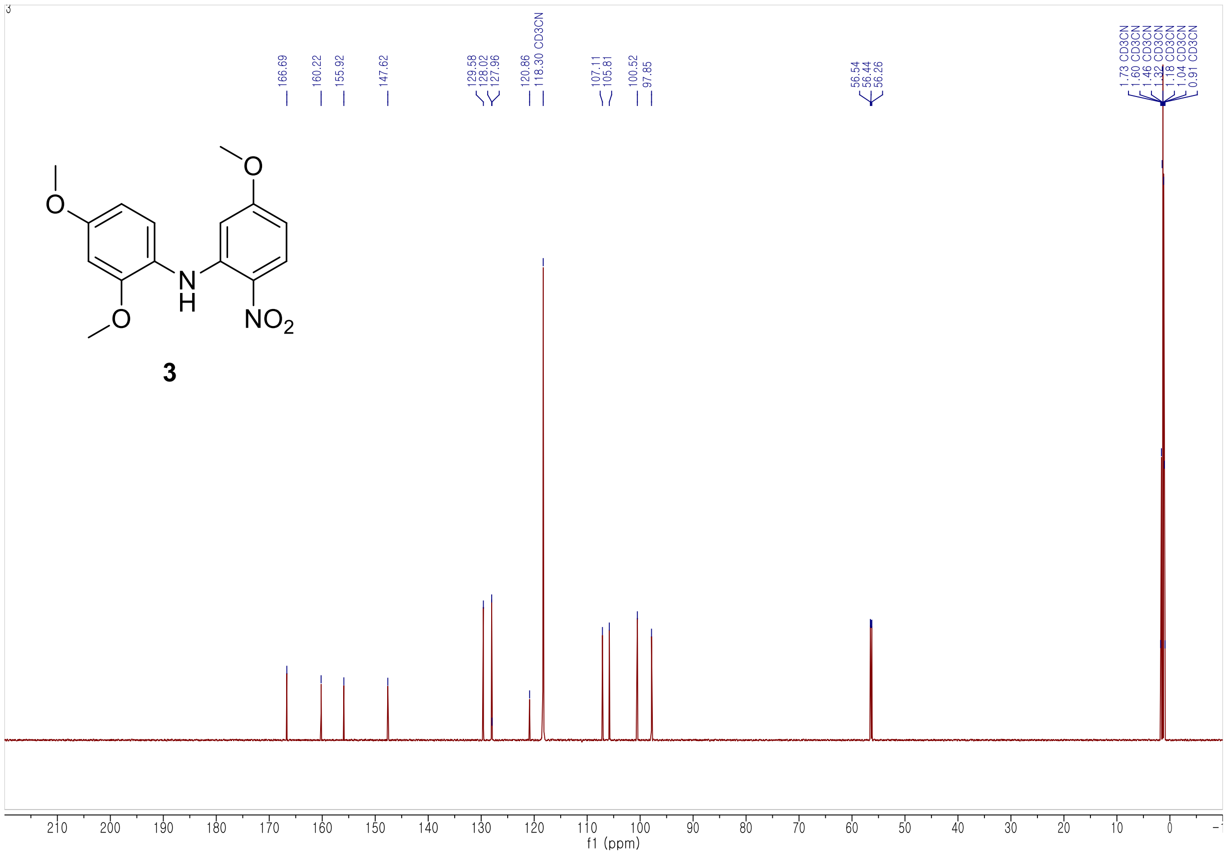

^{1}H NMR (600 MHz, Acetone-d_6) and ^{13}C NMR (150 MHz, Acetone-d_6) spectra of compound **4e**

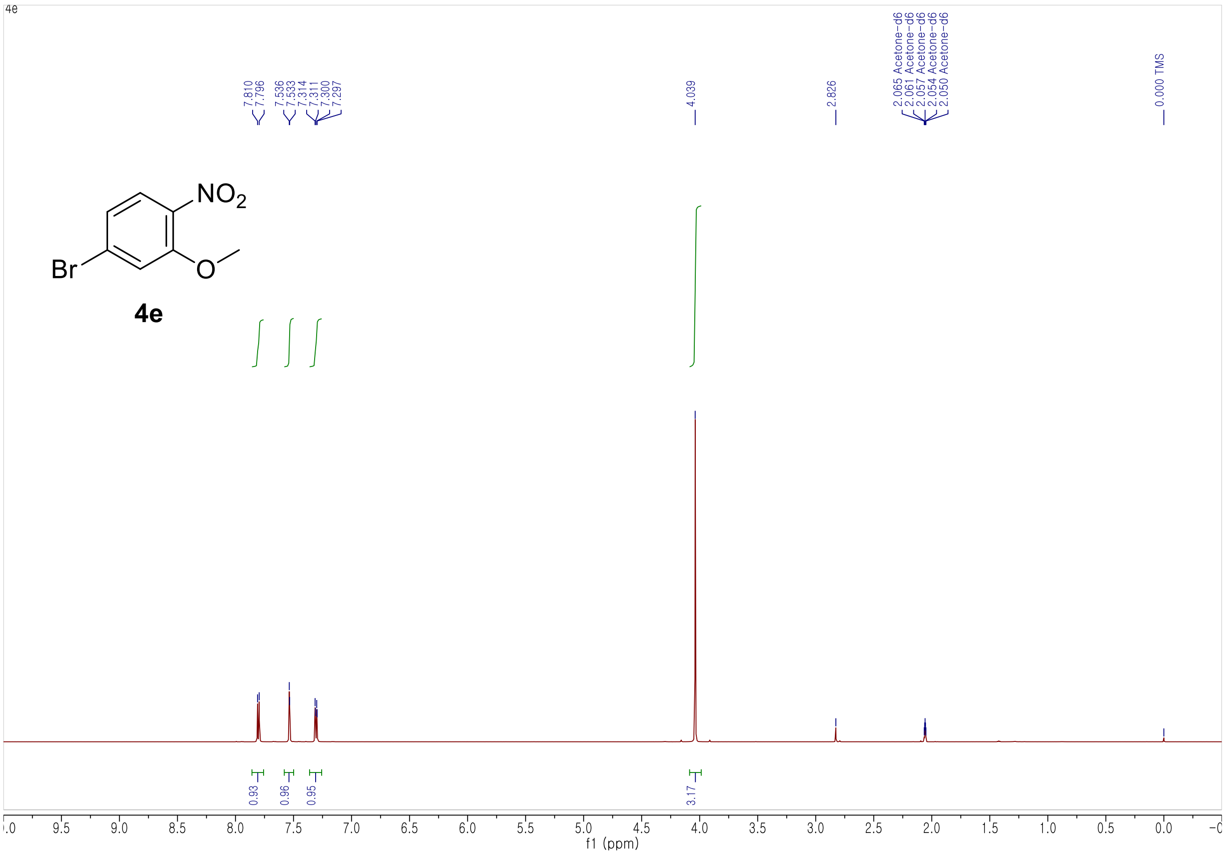

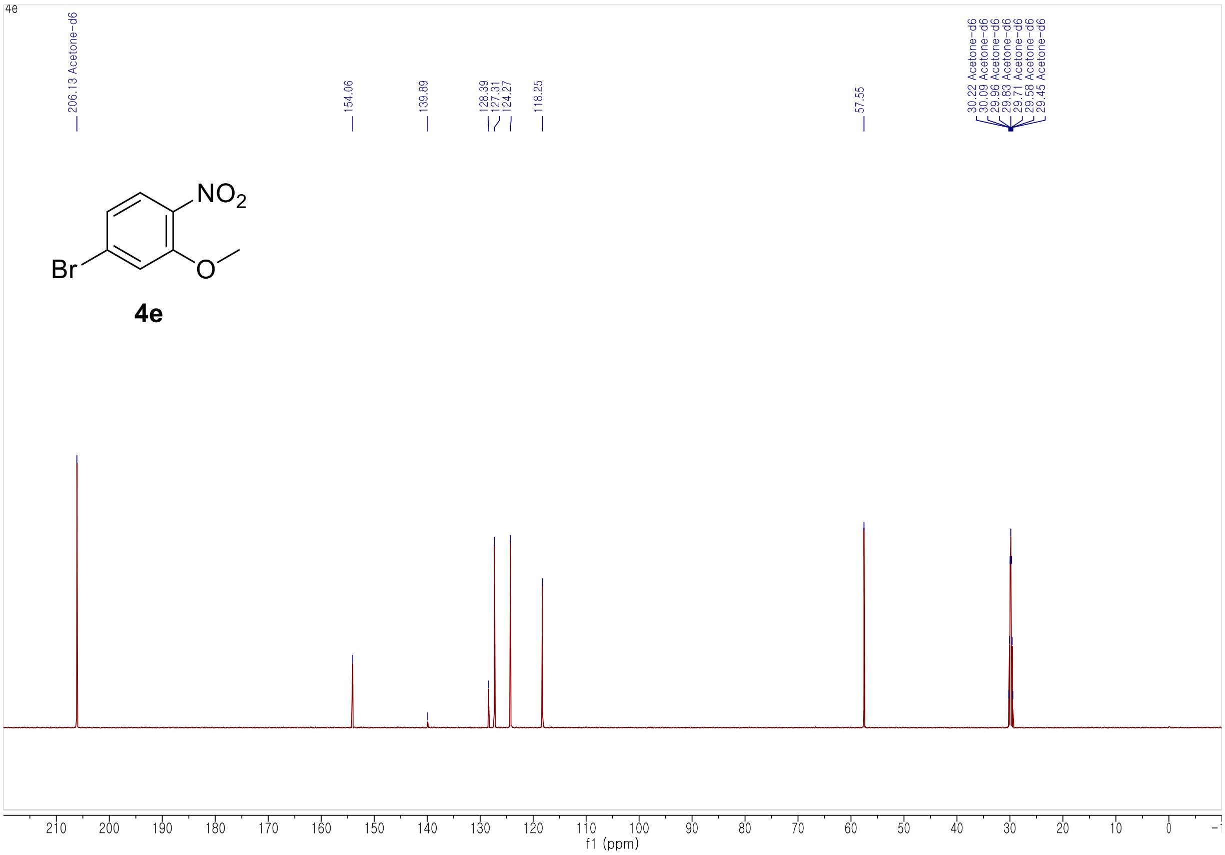

^{1}H NMR (600 MHz, Acetone-d_6) and ^{13}C NMR (150 MHz, Acetone-d_6) spectra of compound **4f**

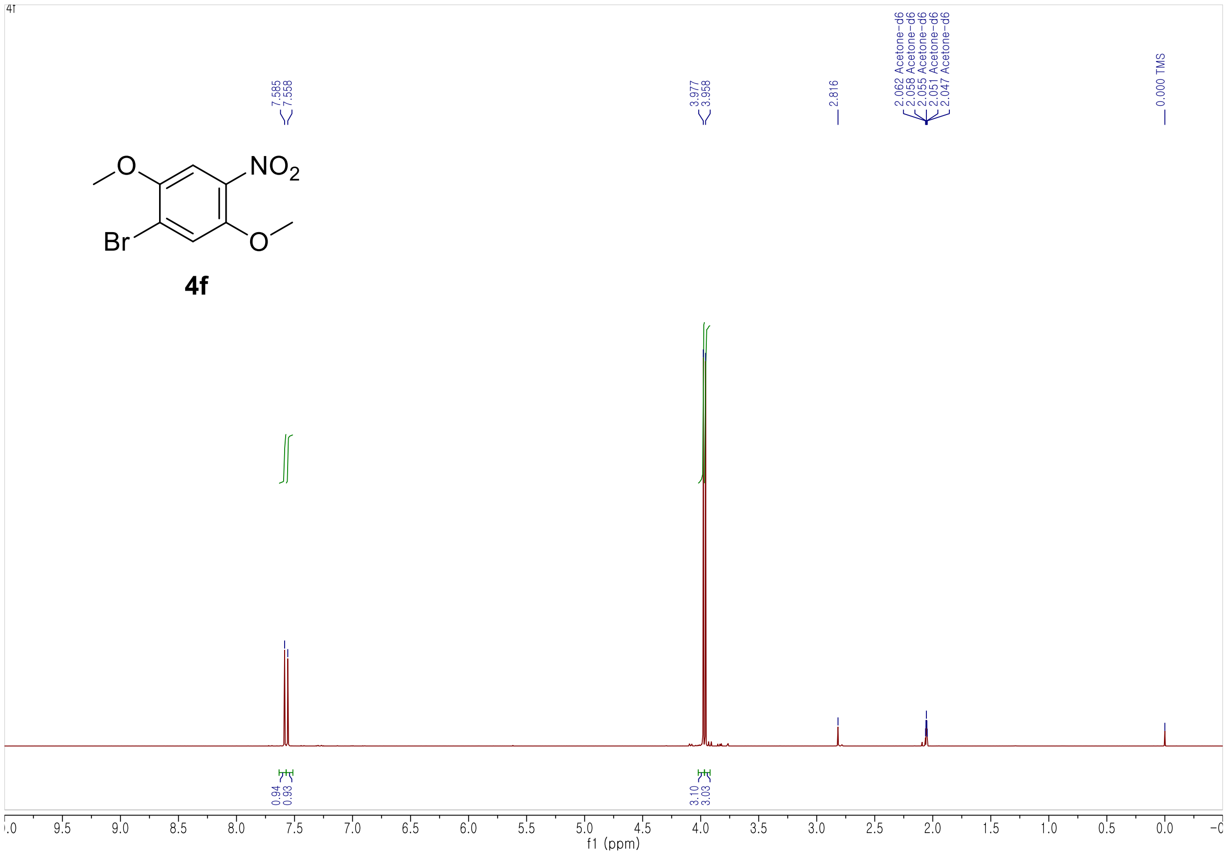

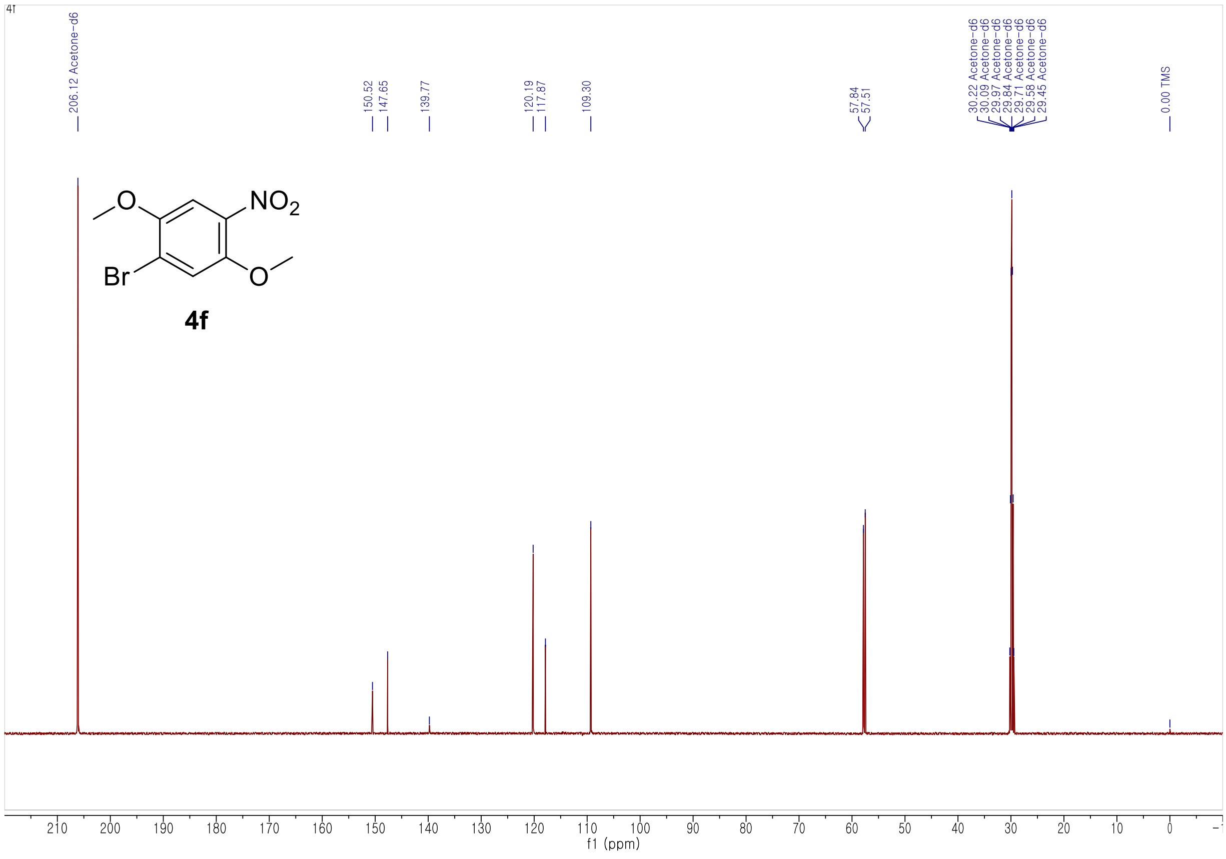

^{1}H NMR (600 MHz, Acetone-d_6) and ^{13}C NMR (150 MHz, Acetone-d_6) spectra of compound **4g**

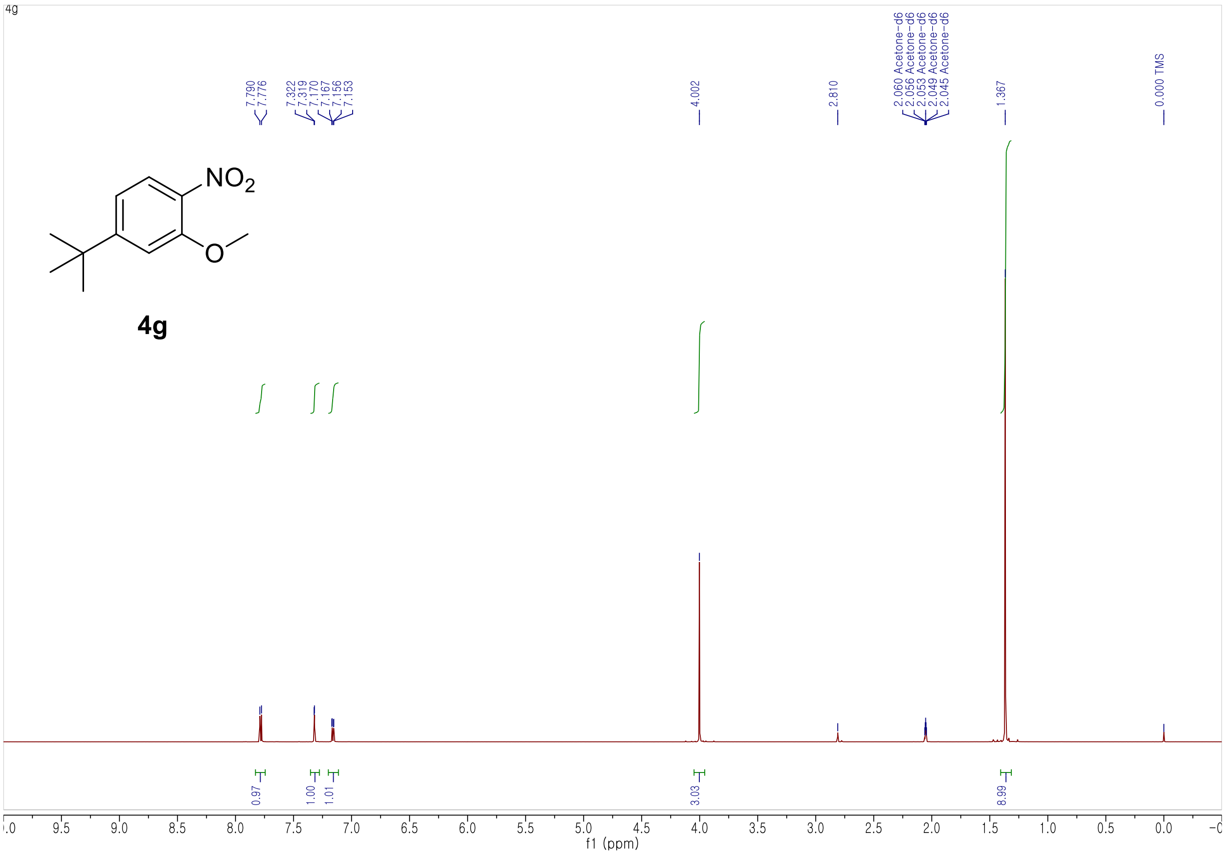

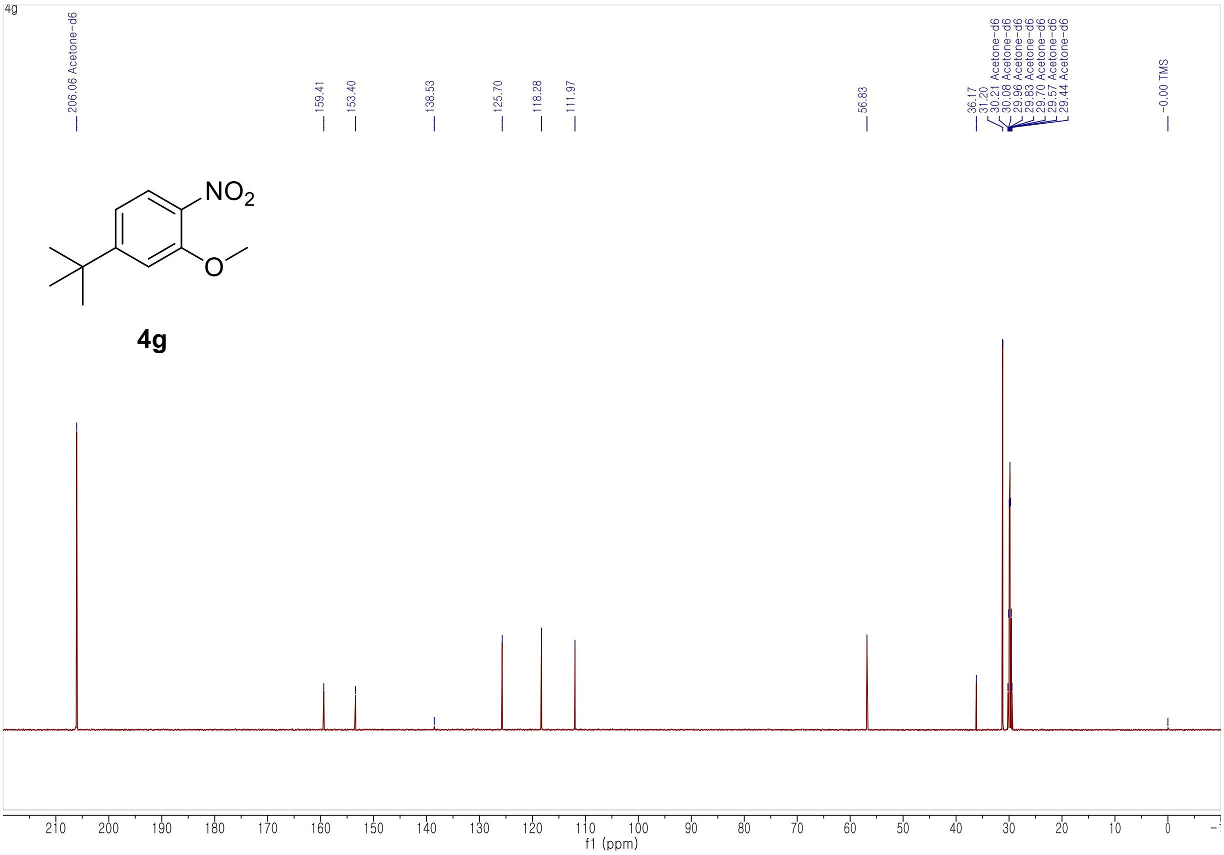

^{1}H NMR (600 MHz, Acetone-d_6) and ^{13}C NMR (150 MHz, Acetone-d_6) spectra of compound **4h**

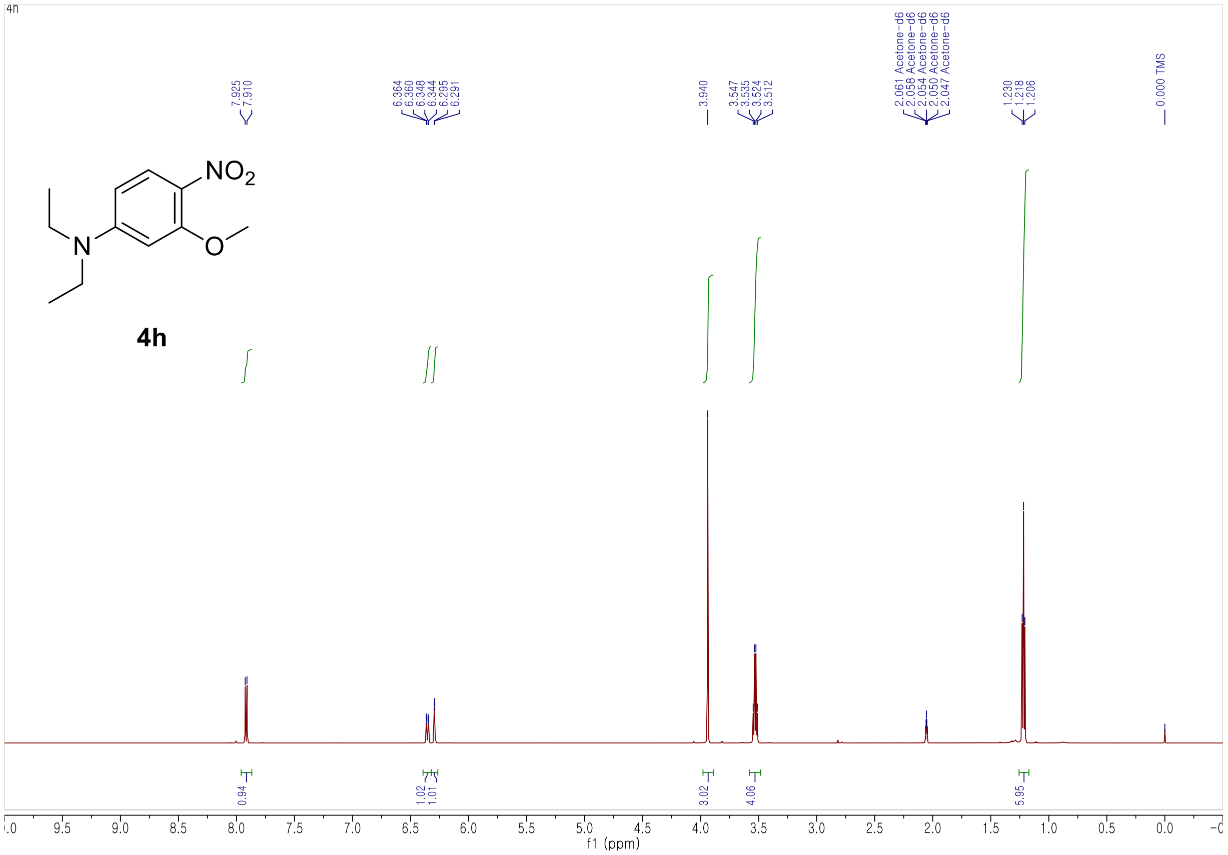

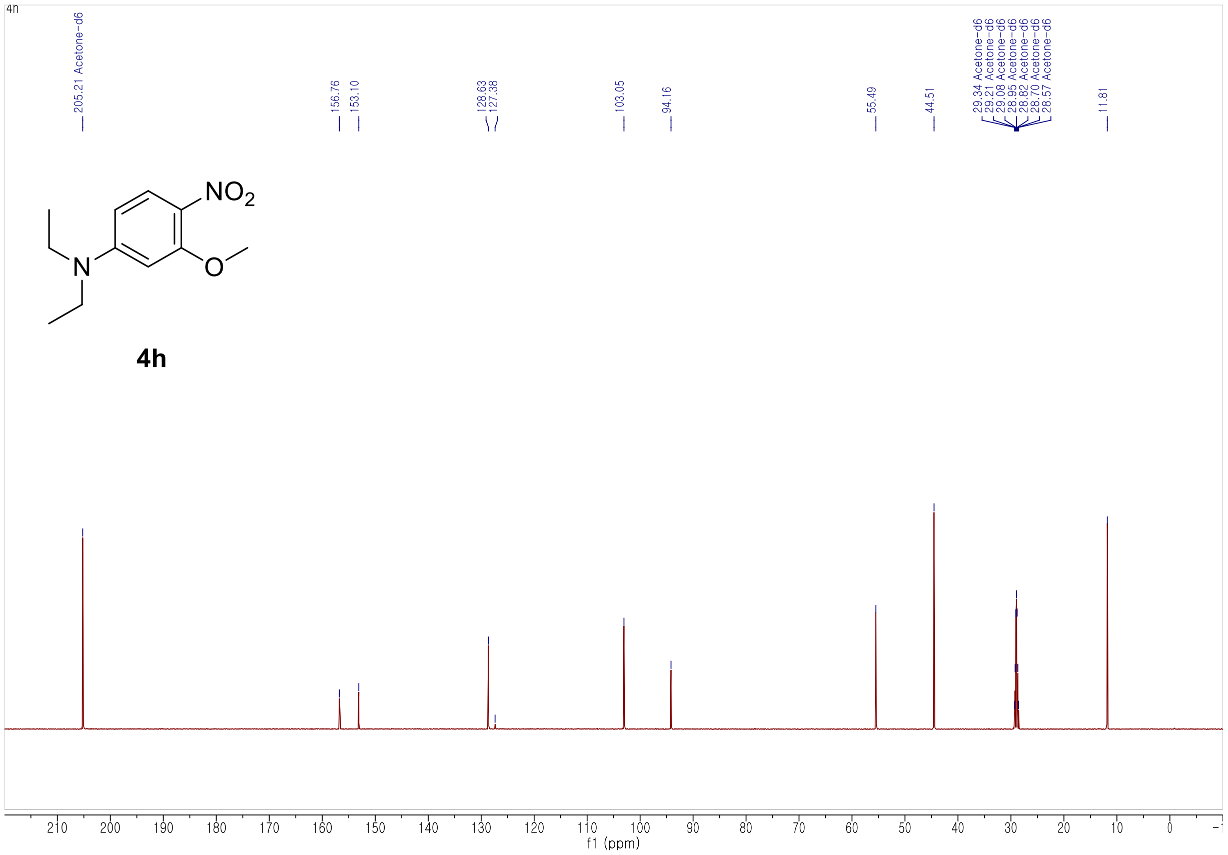

^1H NMR (600 MHz, Acetone-d_6) and ^{13}C NMR (150 MHz, Acetone-d_6) spectra of compound **4i**

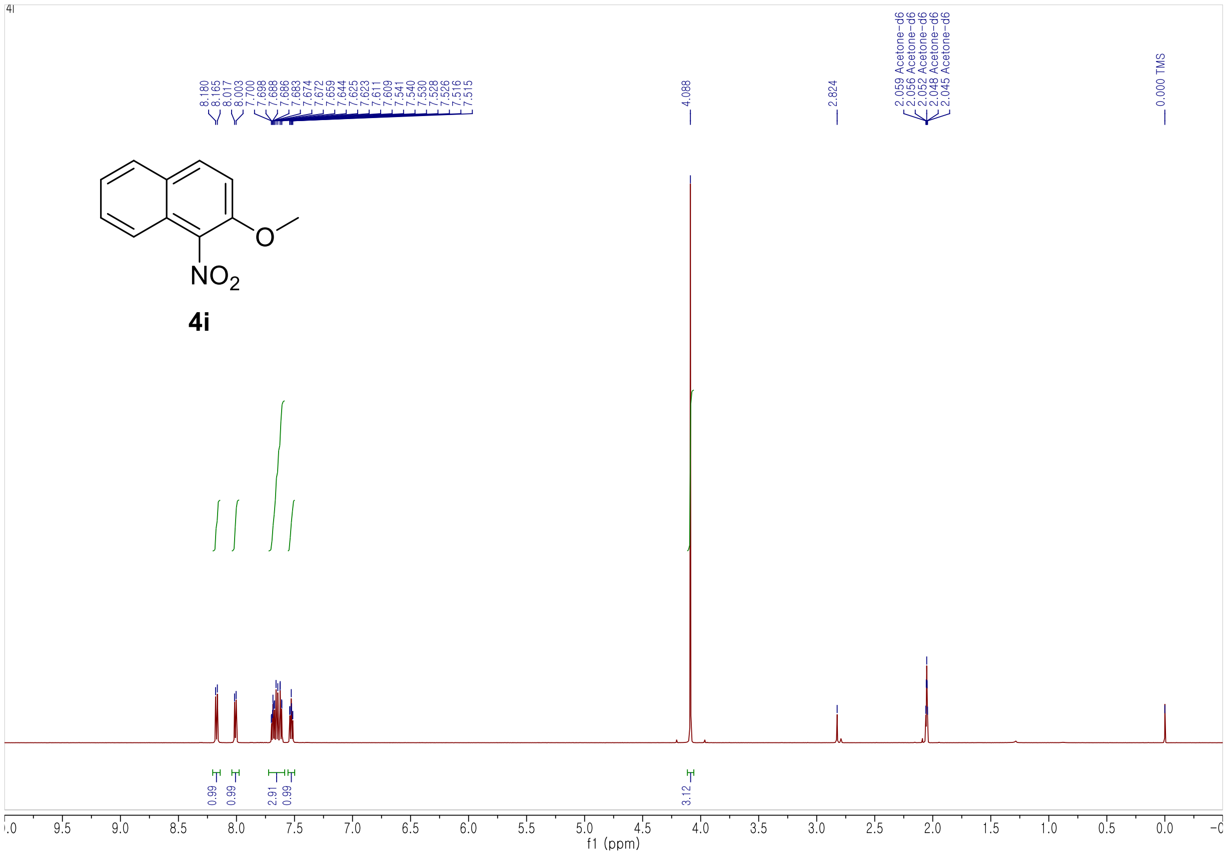

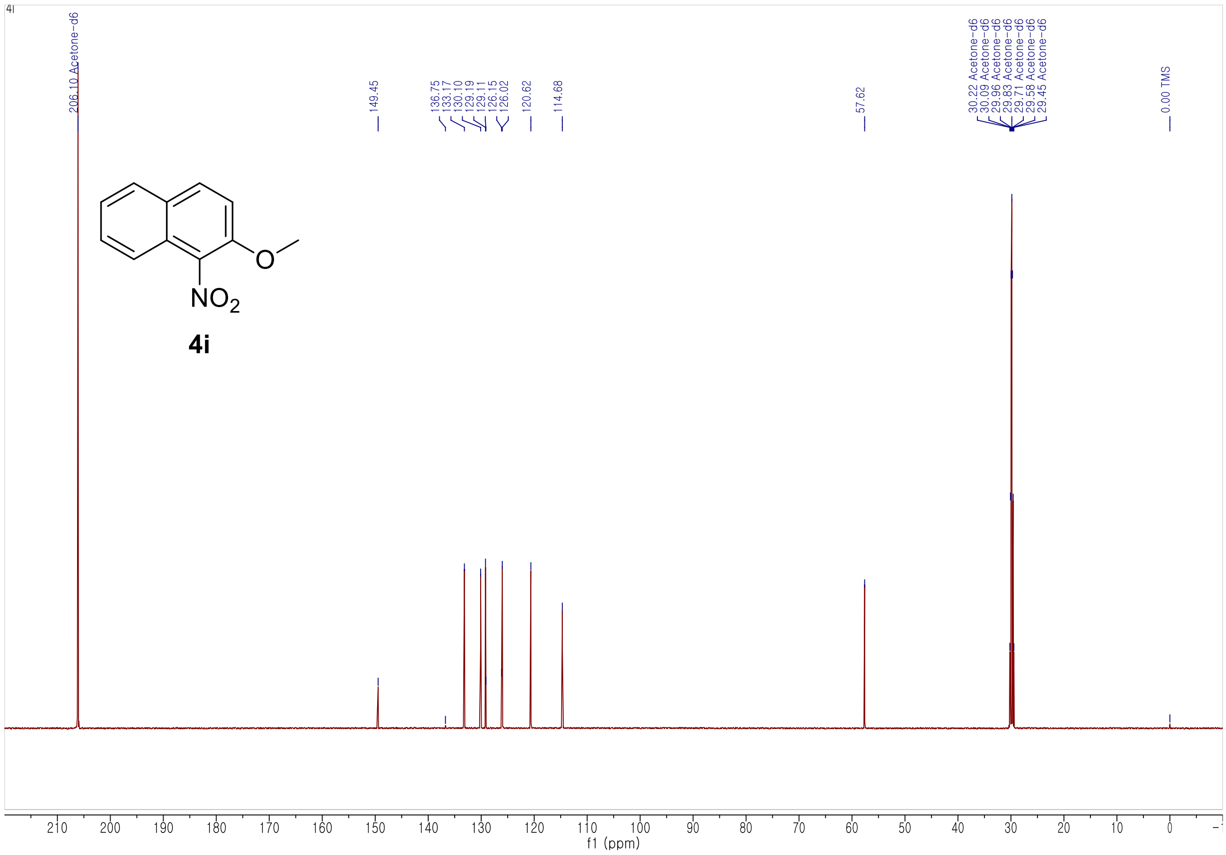

^{1}H NMR (600 MHz, $CDCl_3$) and ^{13}C NMR (150 MHz, $CDCl_3$) spectra of compound **5a**

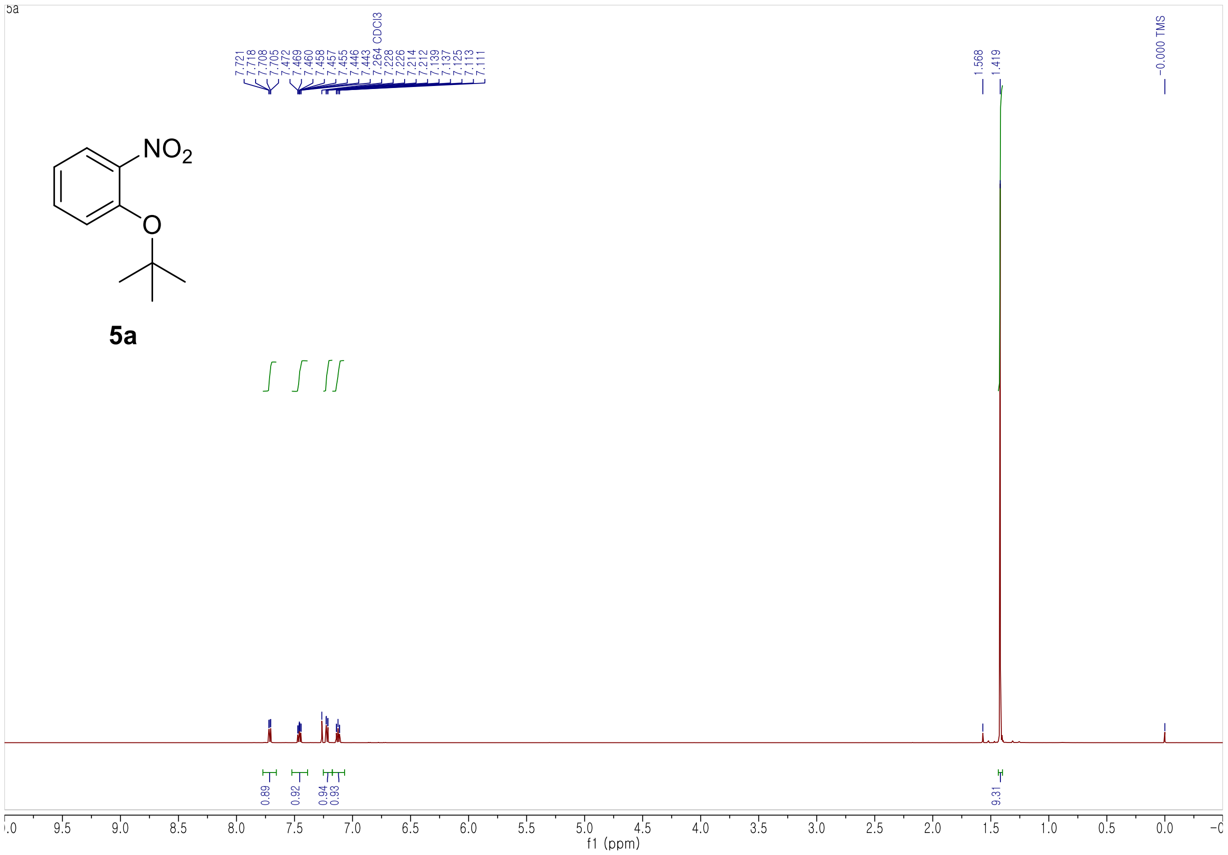

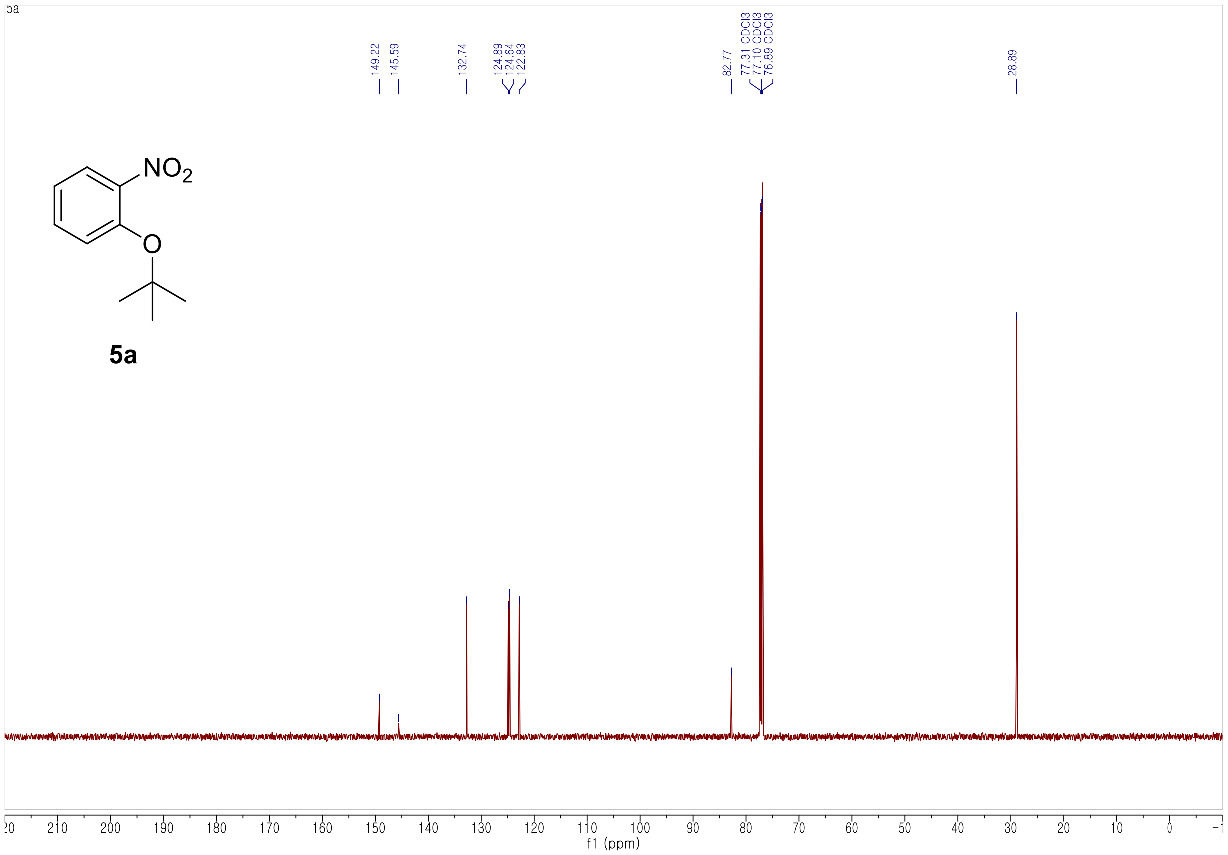

^{1}H NMR (600 MHz, $CDCl_3$) and ^{13}C NMR (150 MHz, $CDCl_3$) spectra of compound **5b**

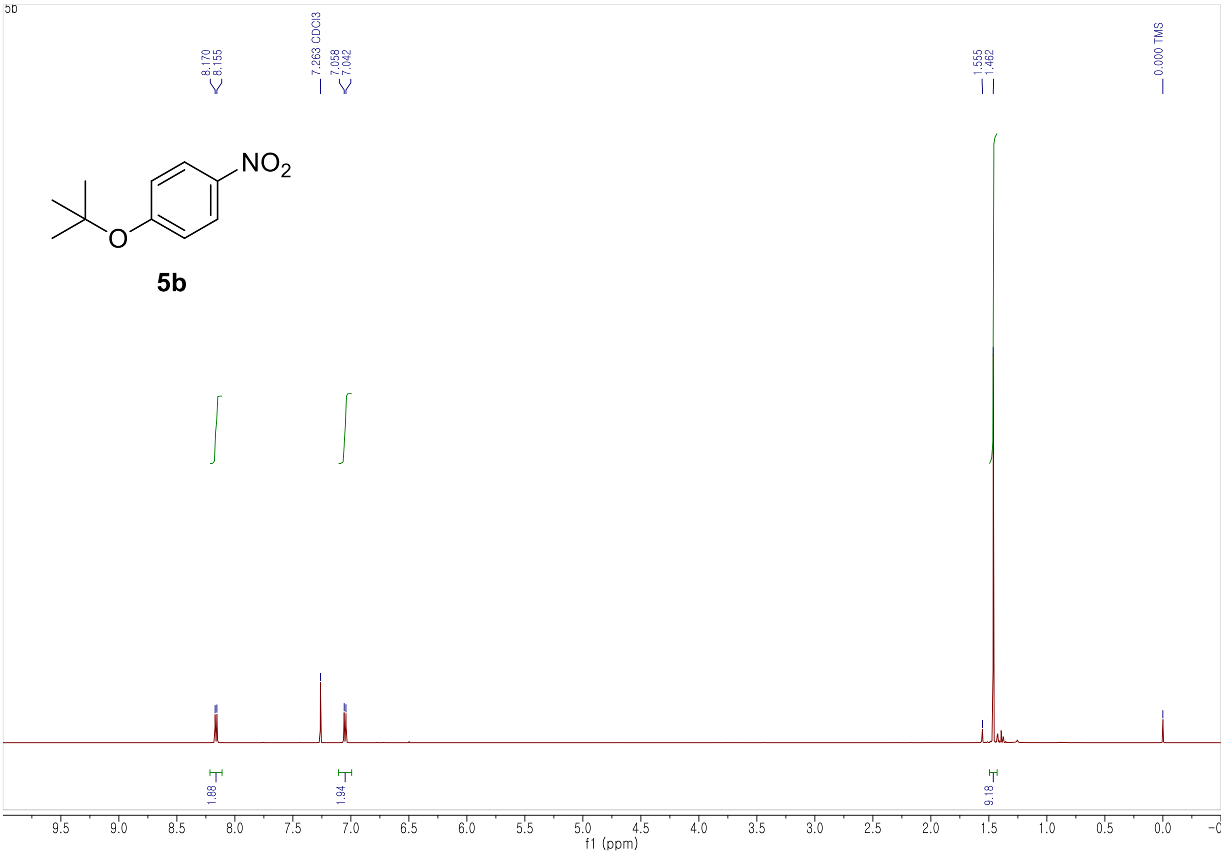

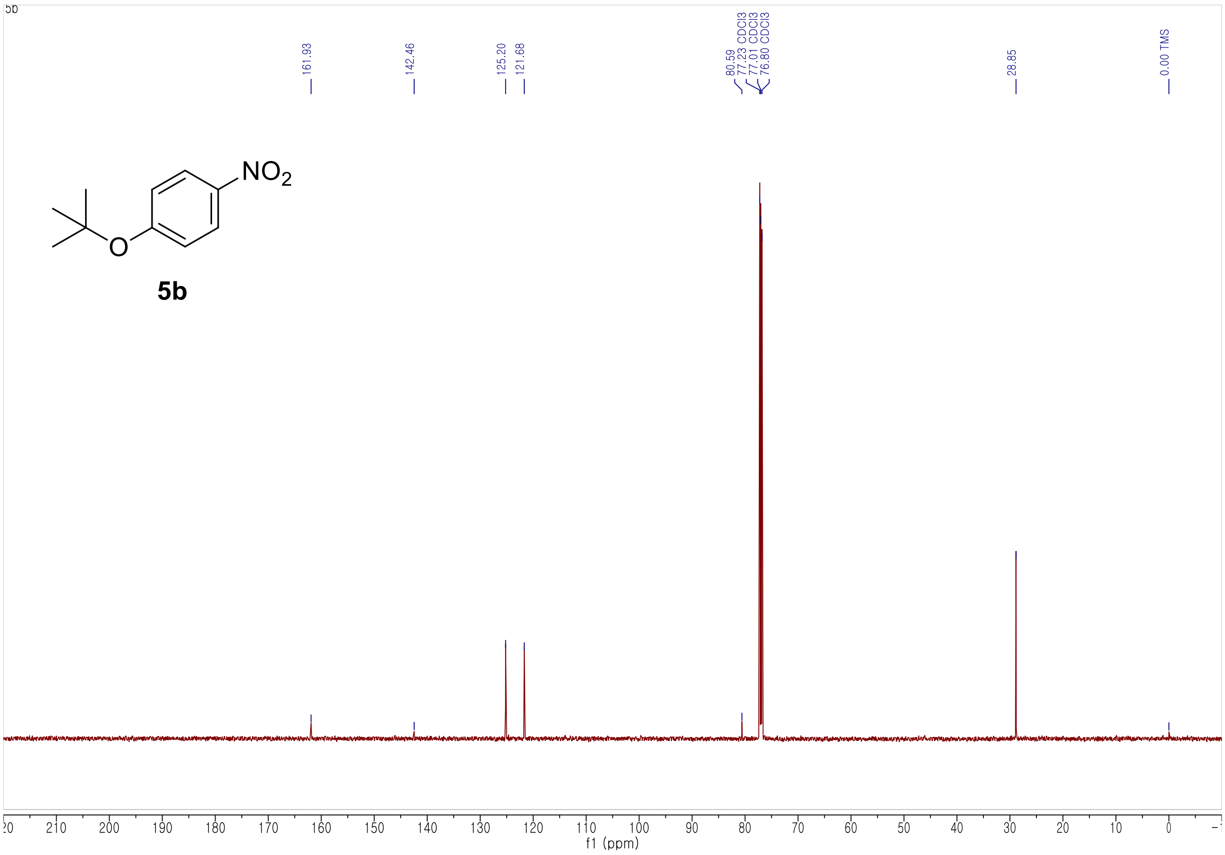

^{1}H NMR (600 MHz, $CDCl_3$) and ^{13}C NMR (150 MHz, $CDCl_3$) spectra of compound **5c**

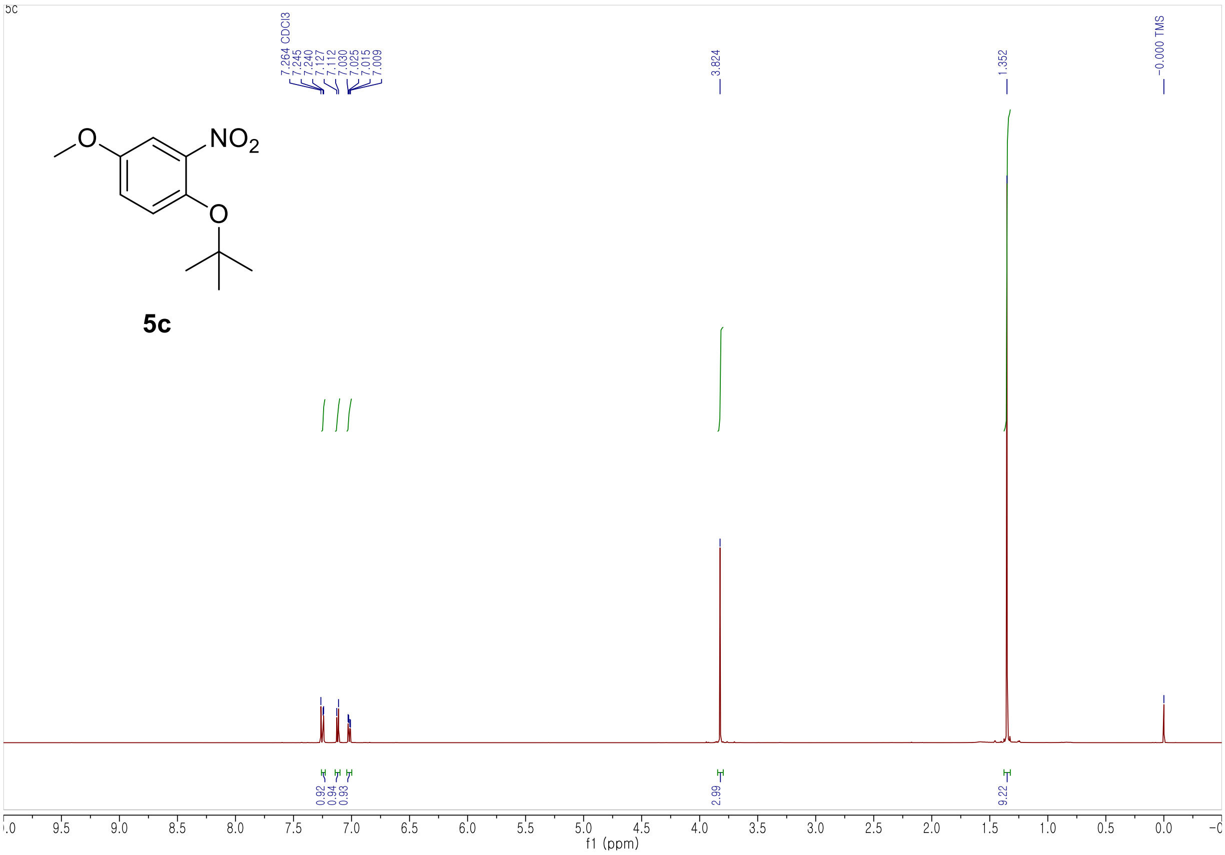

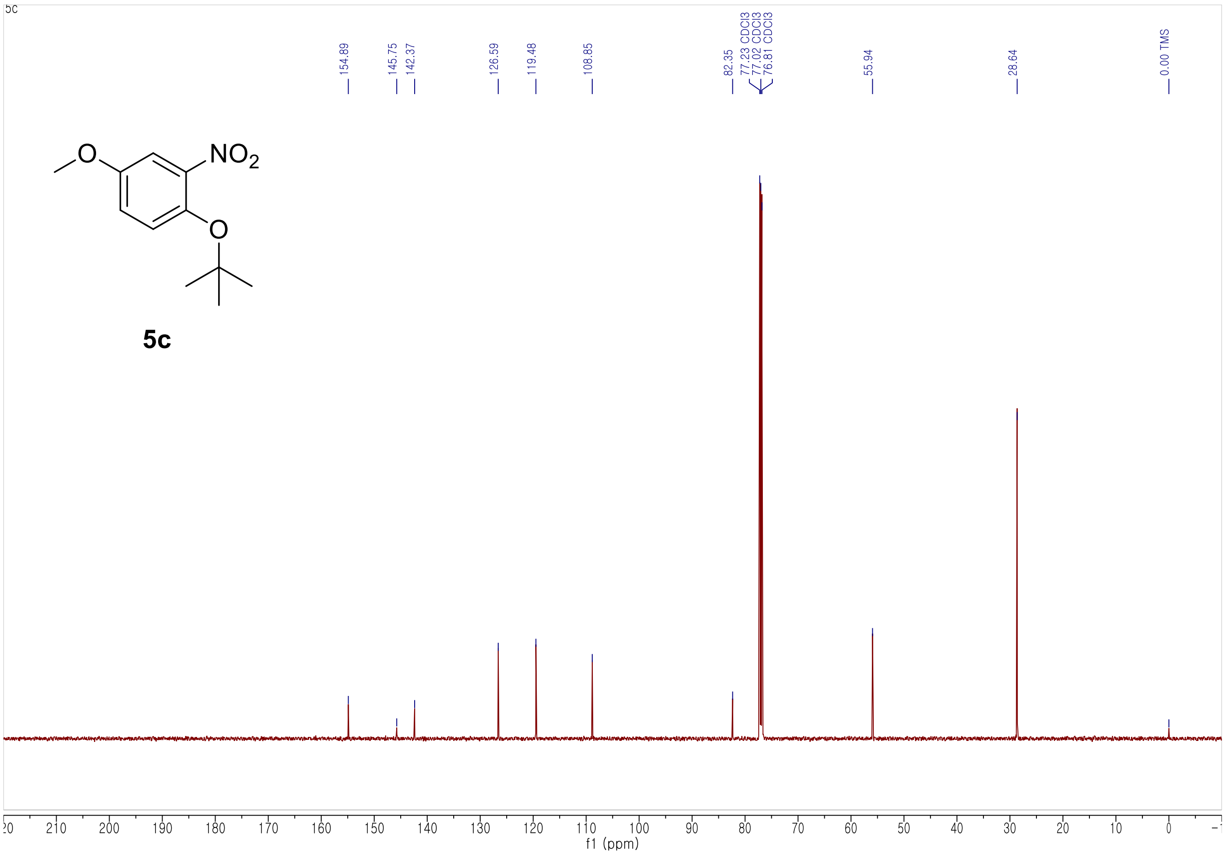

^{1}H NMR (600 MHz, CD_3CN) and ^{13}C NMR (150 MHz, CD_3CN) spectra of compound **5d**

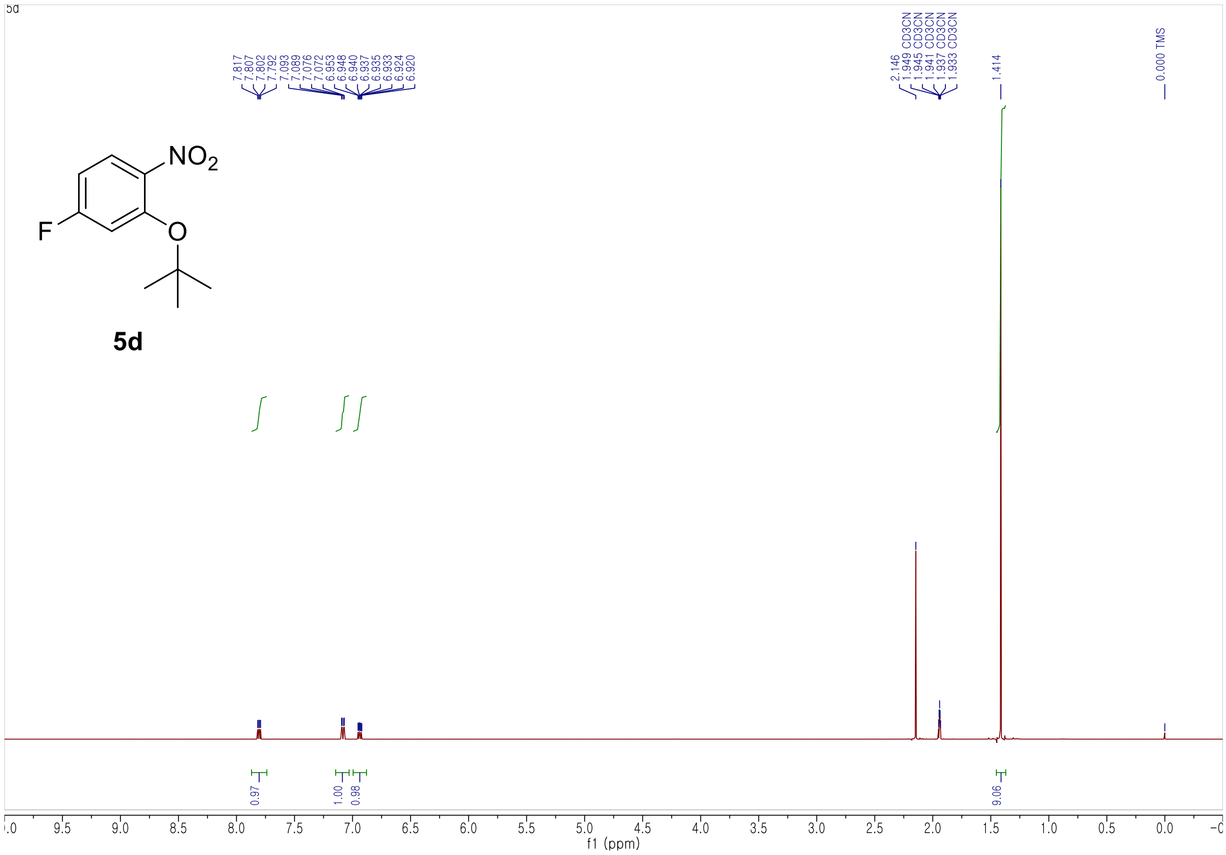

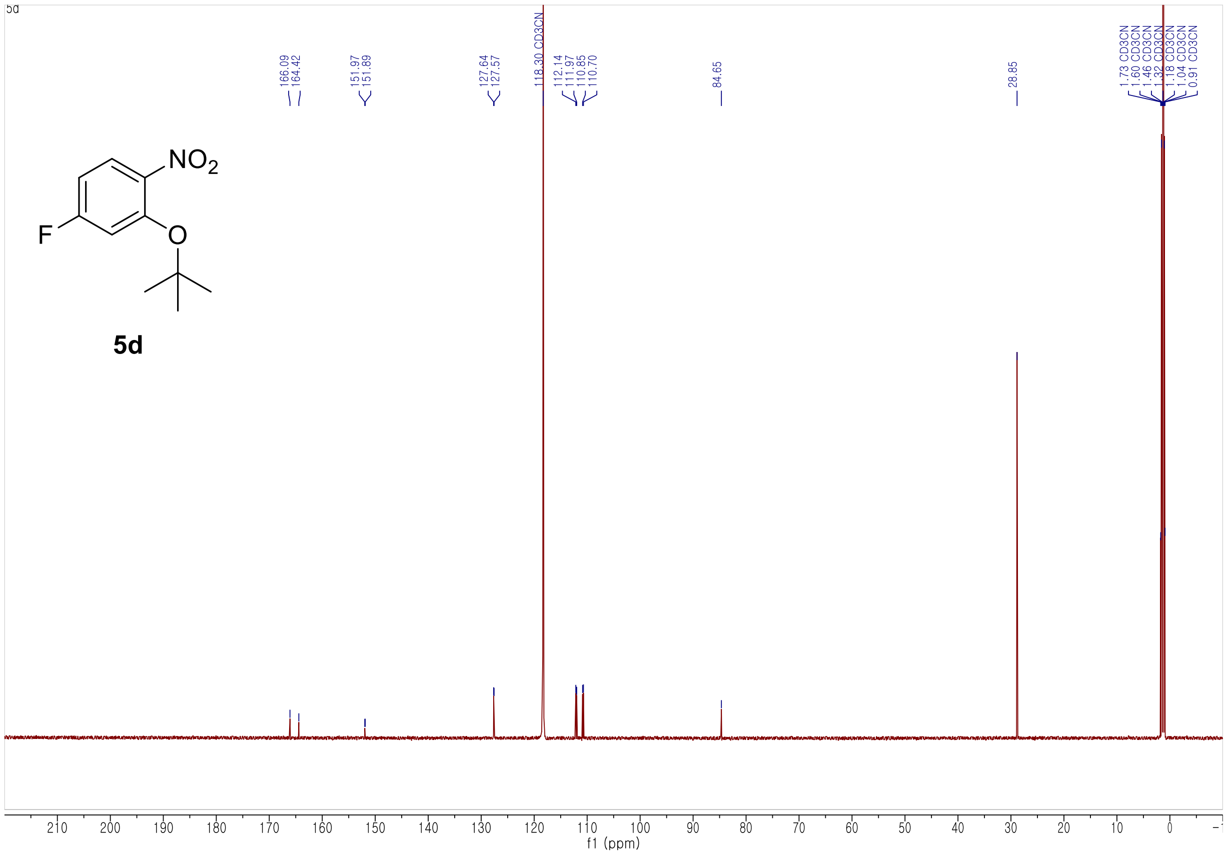

^{1}H NMR (600 MHz, $CDCl_3$) and ^{13}C NMR (150 MHz, CD_3CN) spectra of compound **5e**

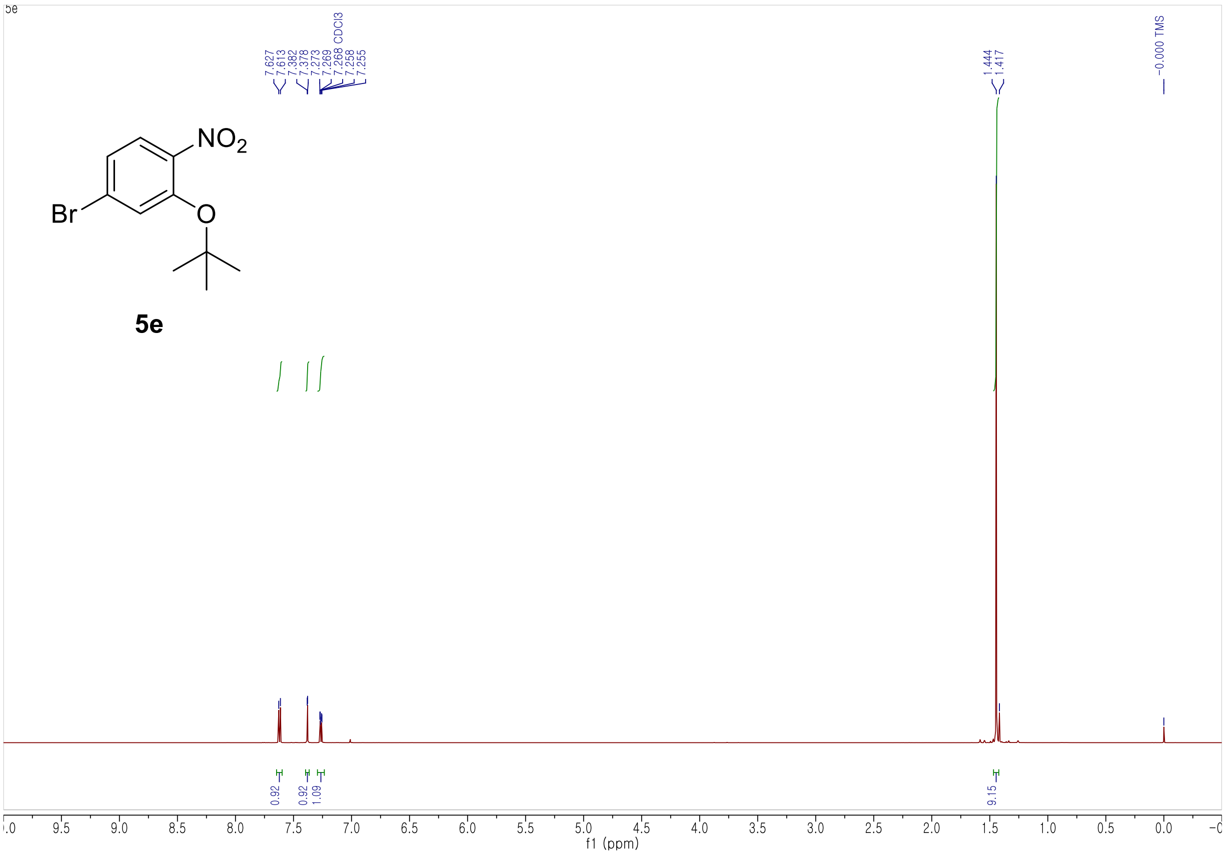

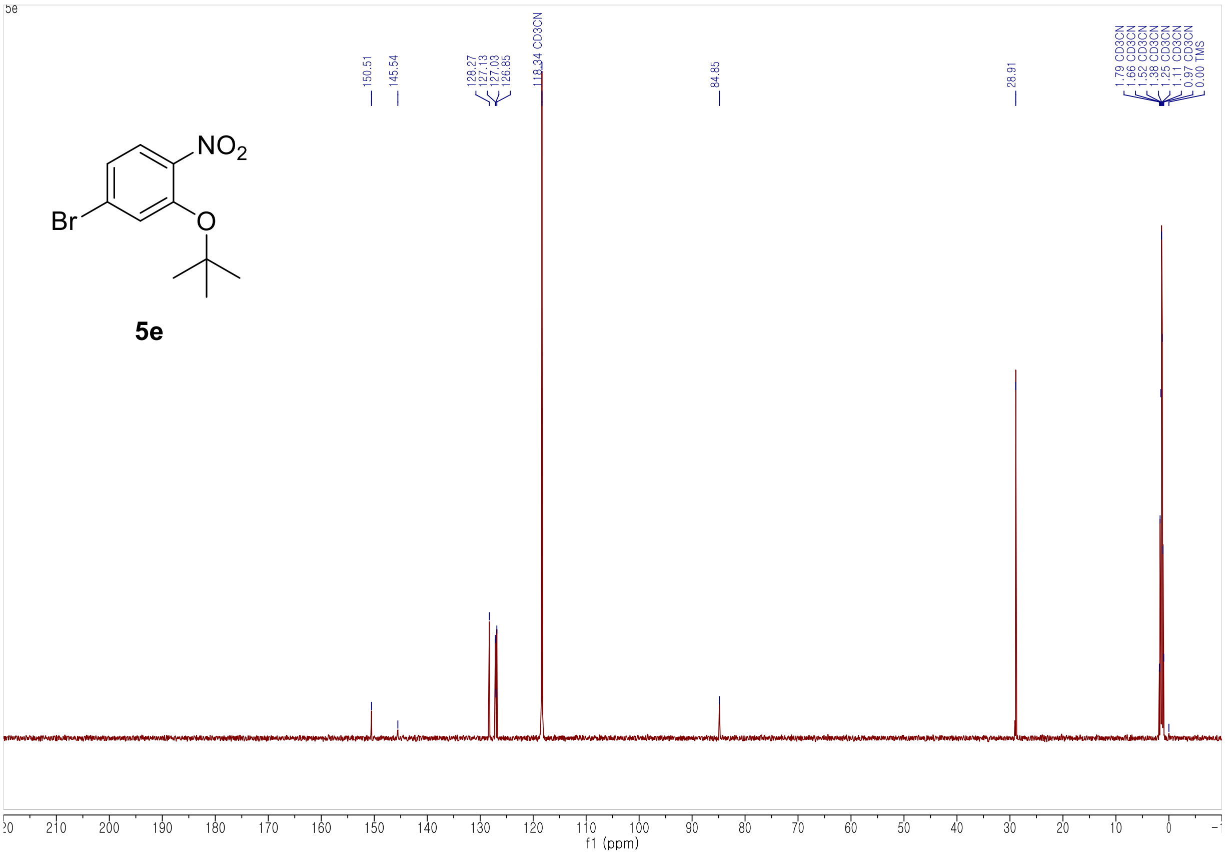

^{1}H NMR (600 MHz, CD_3CN) and ^{13}C NMR (150 MHz, CD_3CN) spectra of compound **5f**

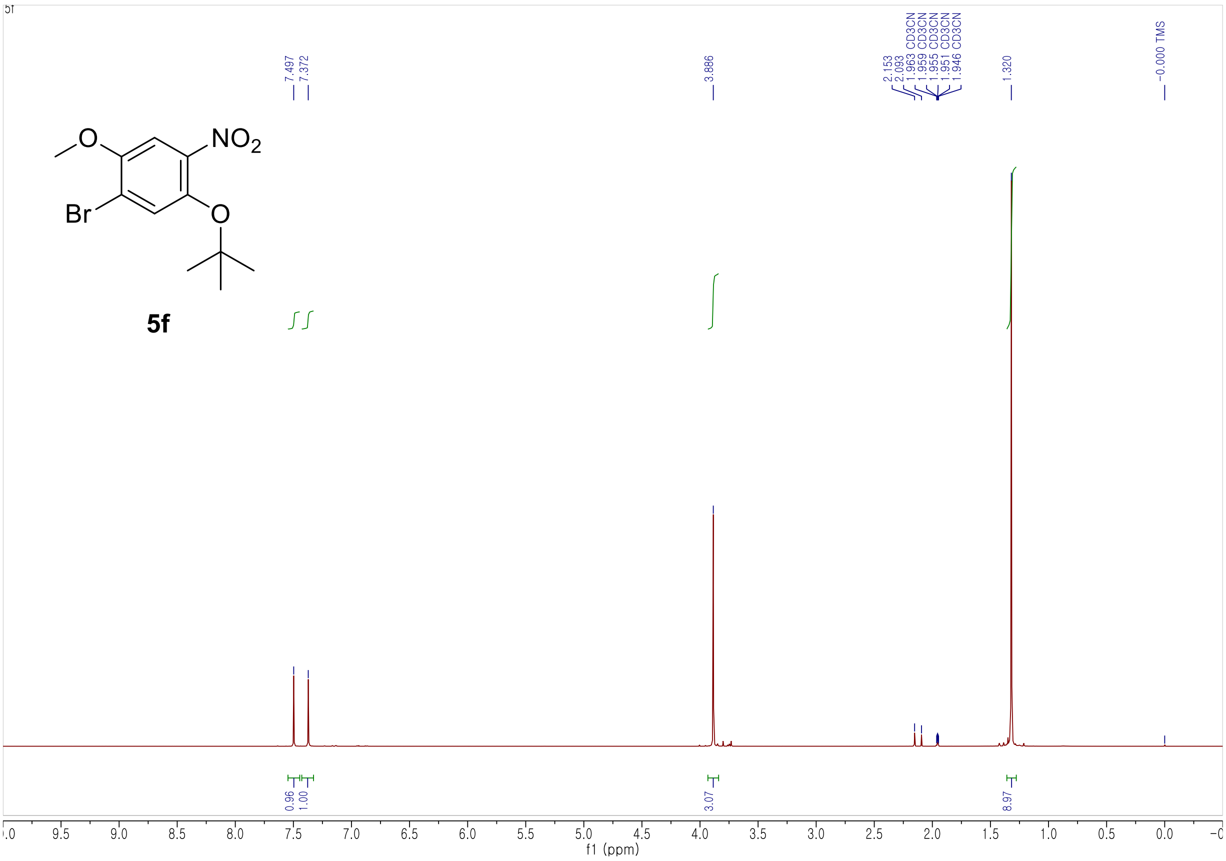

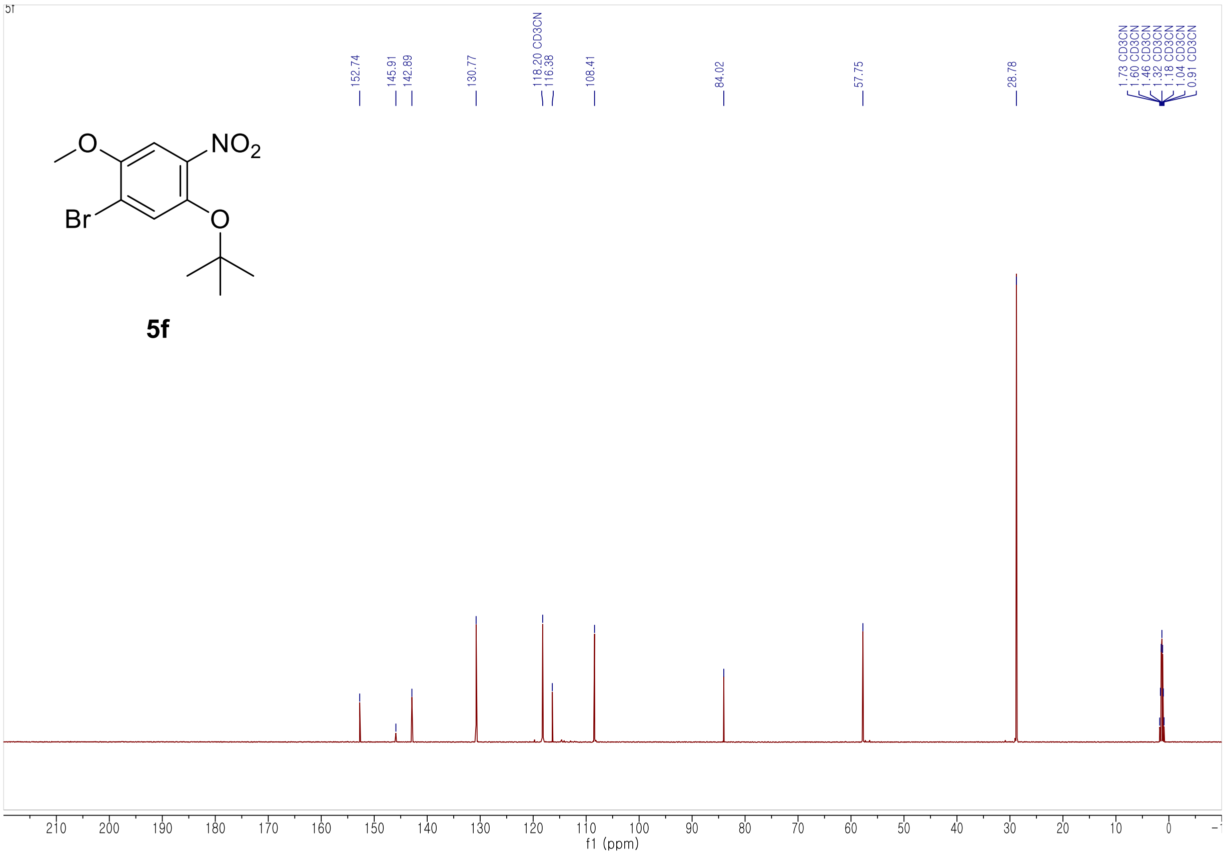

^{1}H NMR (600 MHz, CD_3CN) and ^{13}C NMR (150 MHz, CD_3CN) spectra of compound **5g**

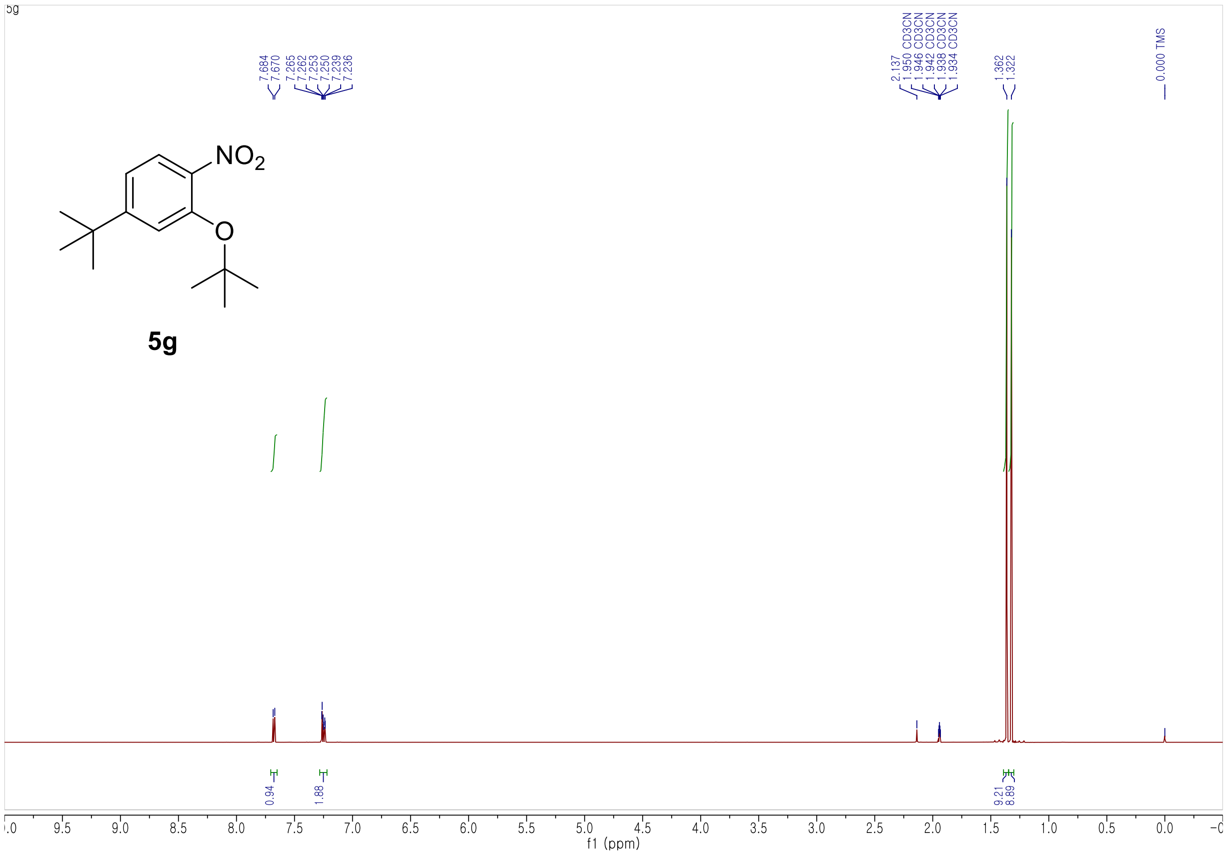

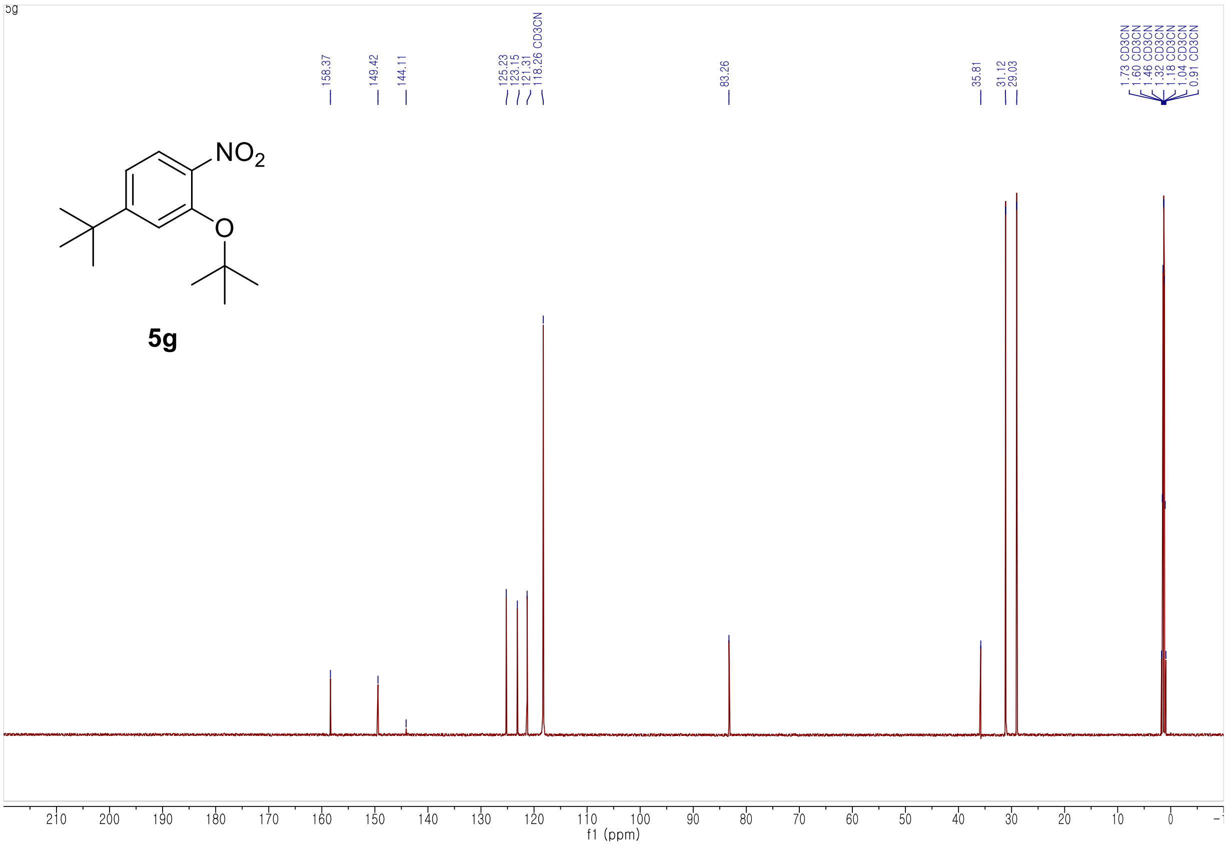

^{1}H NMR (600 MHz, Acetone-d_6) and ^{13}C NMR (150 MHz, Acetone-d_6) spectra of compound **5h**

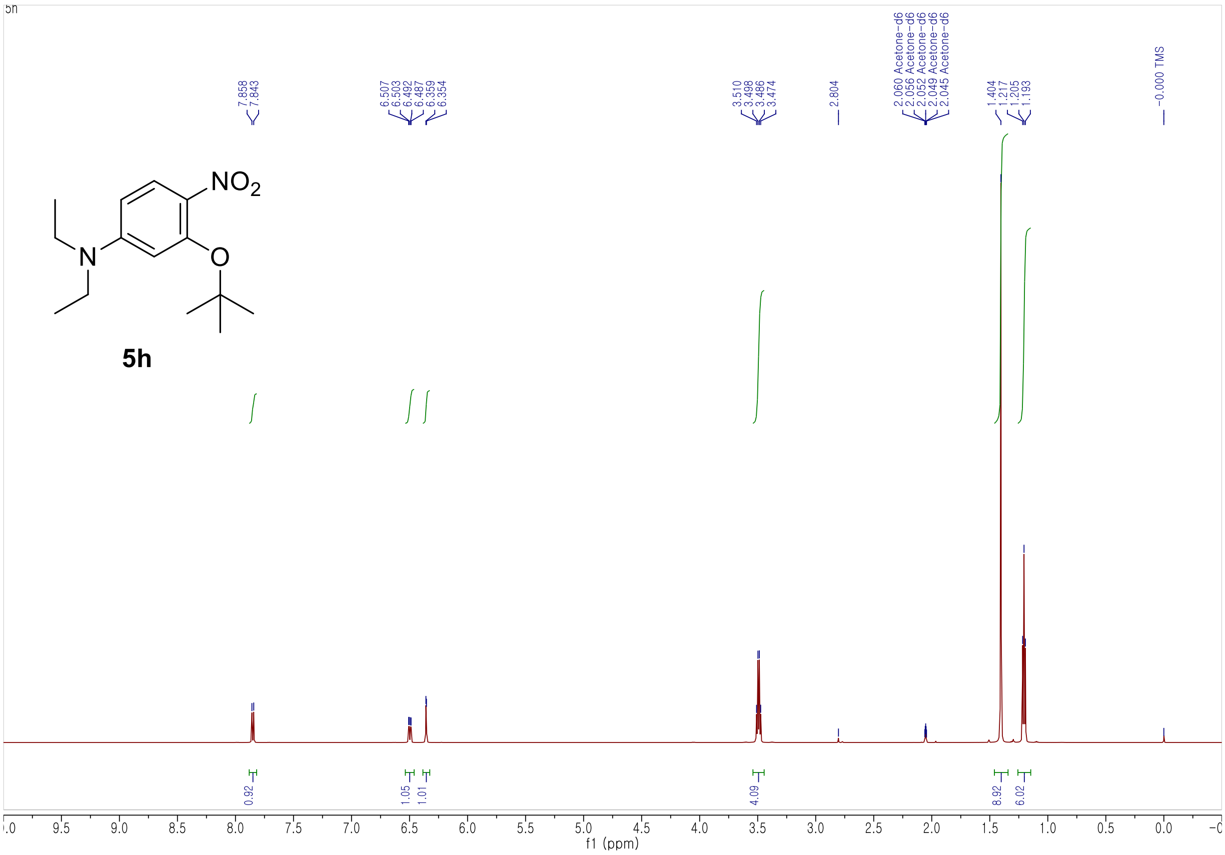

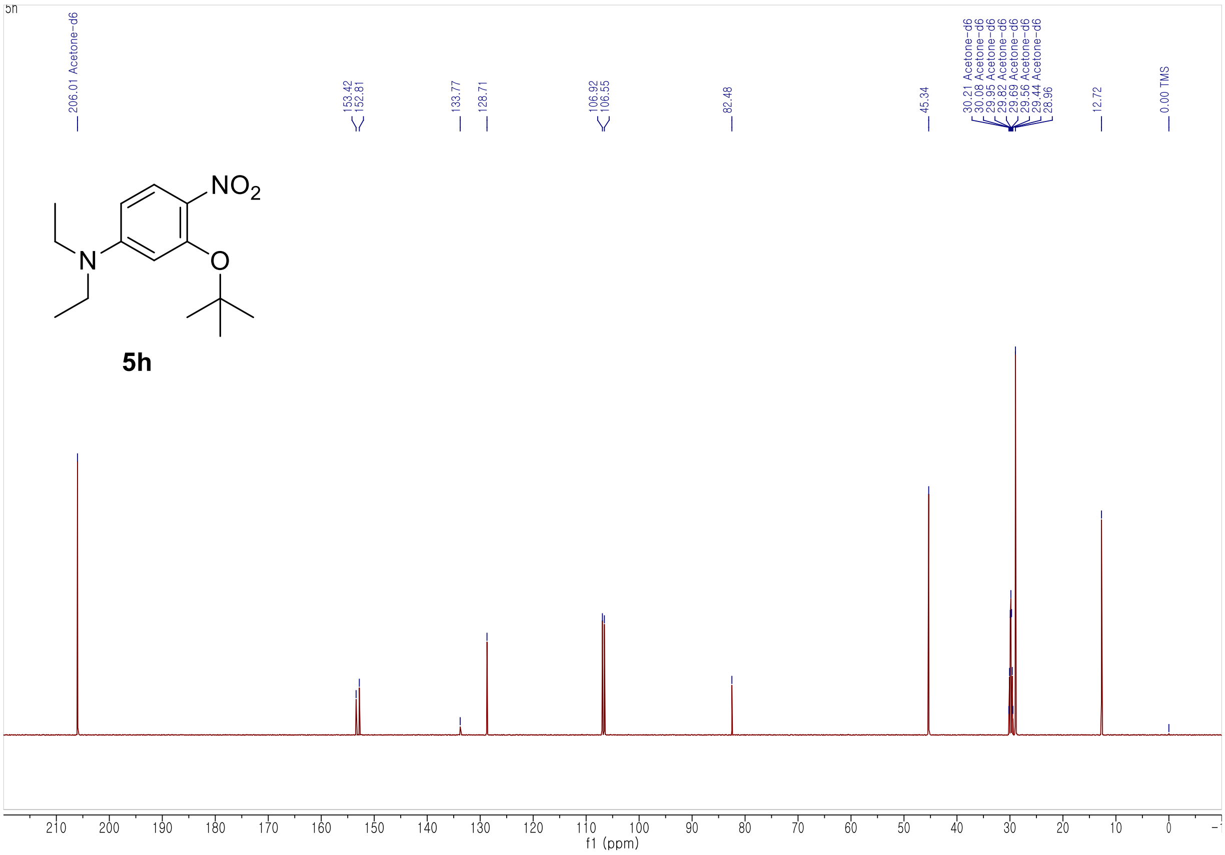

^{1}H NMR (600 MHz, $CDCl_3$) and ^{13}C NMR (150 MHz, $CDCl_3$) spectra of compound **5i**

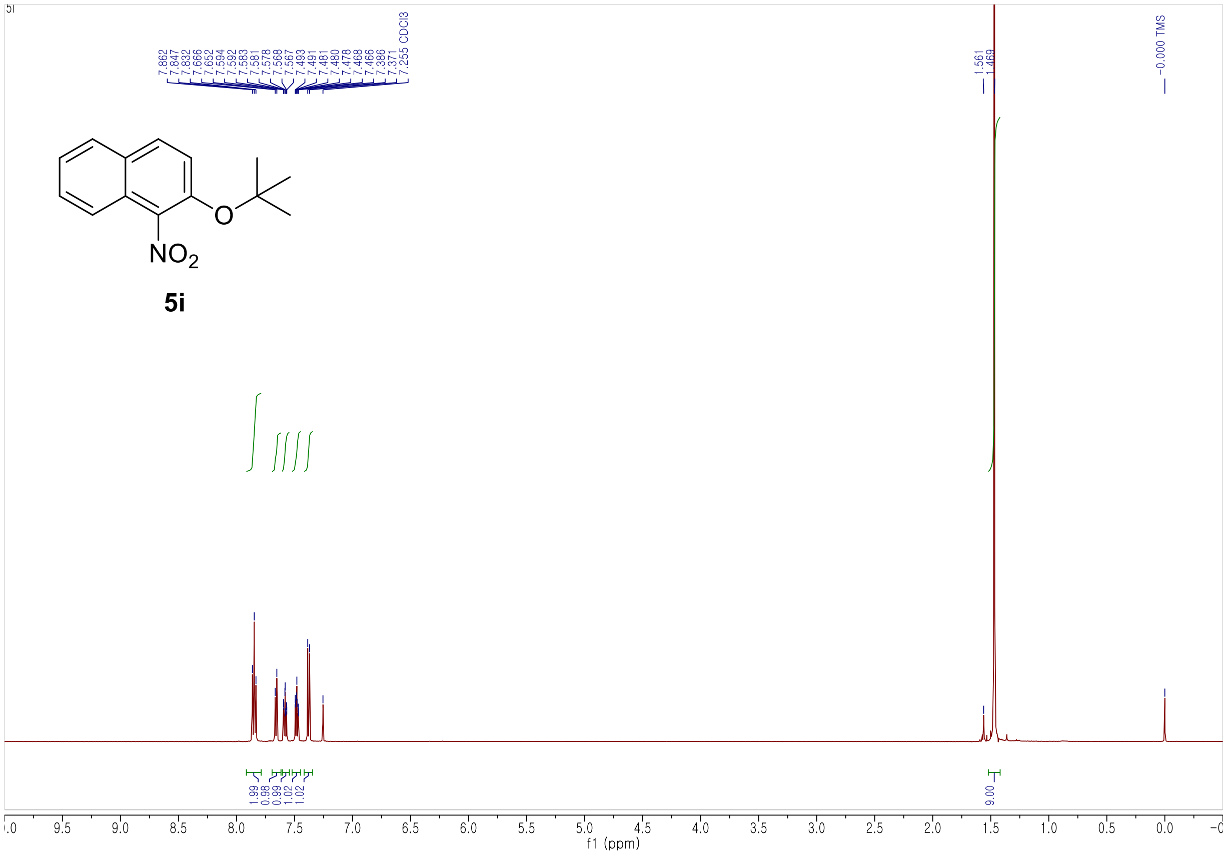

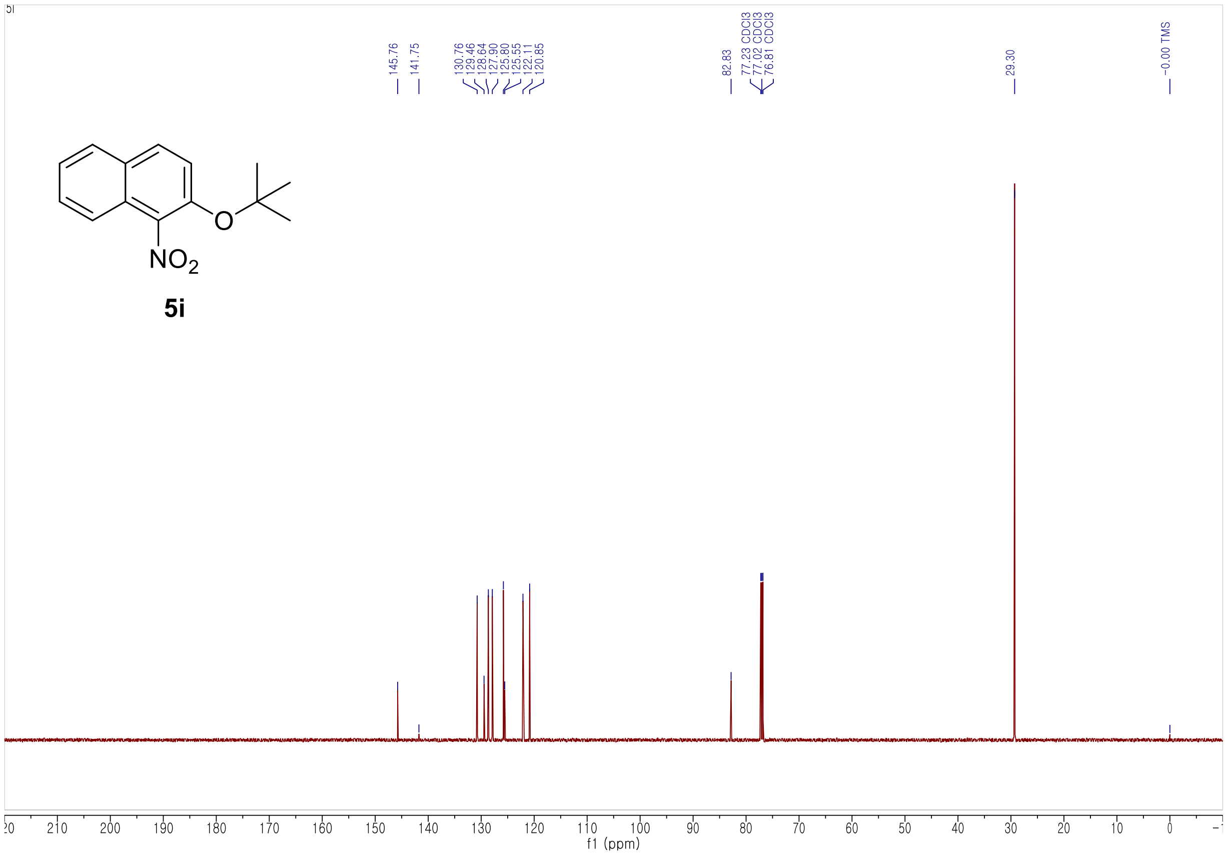

^{1}H NMR (600 MHz, $CDCl_3$) and ^{13}C NMR (150 MHz, $CDCl_3$) spectra of compound **6**

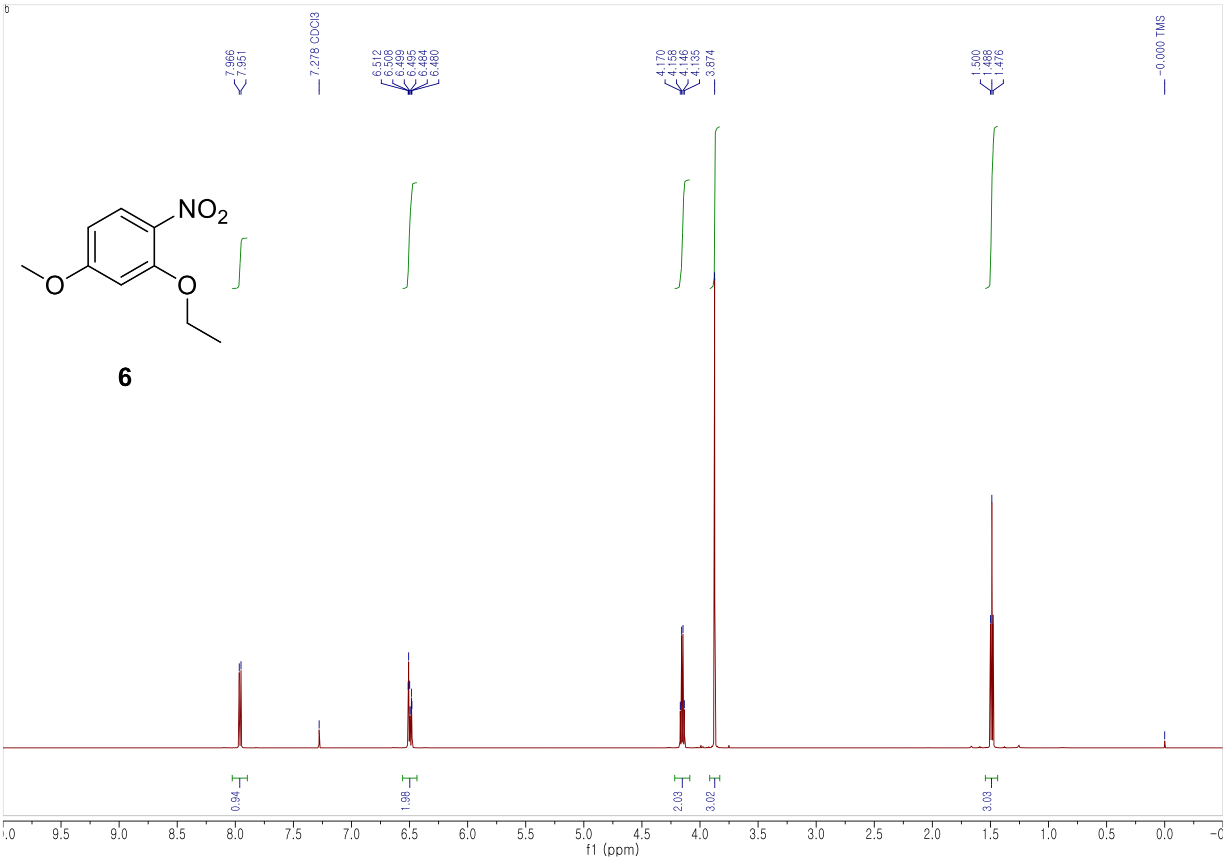

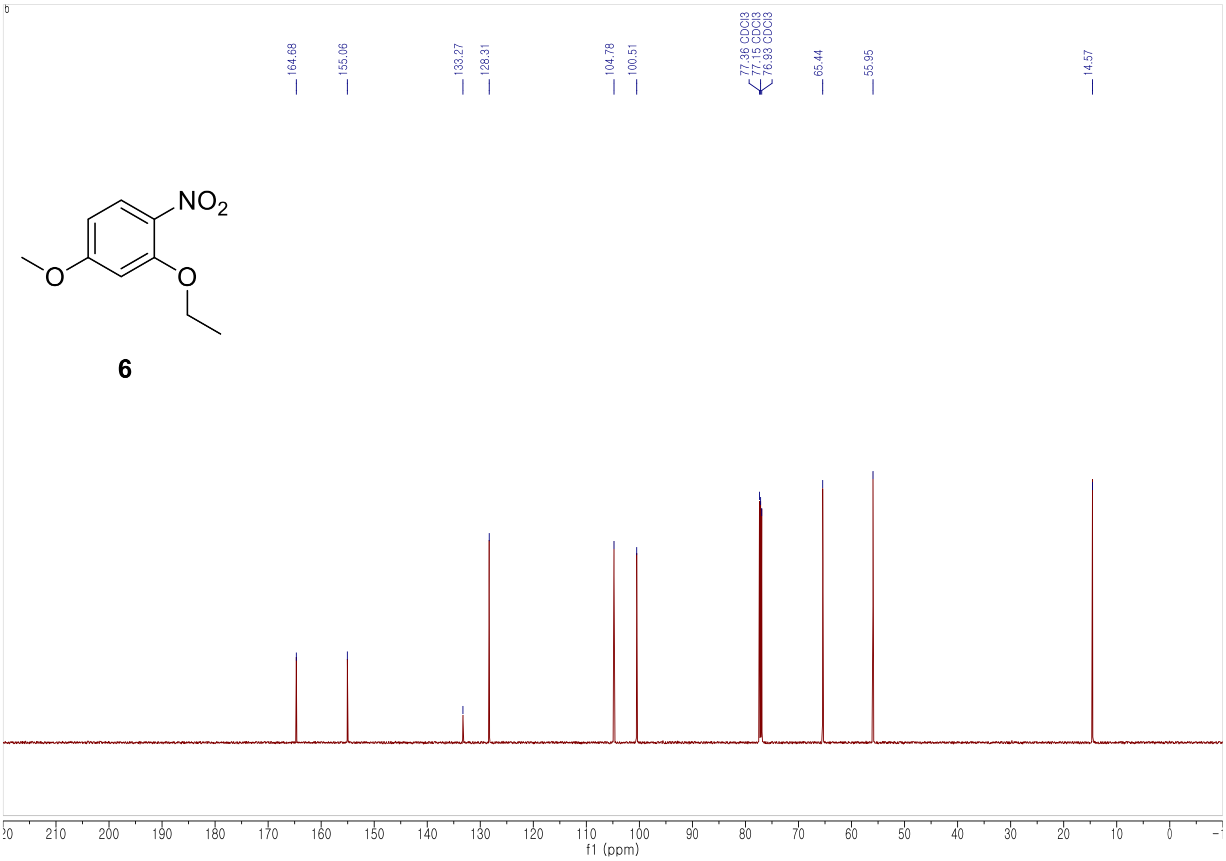

^{1}H NMR (600 MHz, $CDCl_3$) and ^{13}C NMR (150 MHz, $CDCl_3$) spectra of compound **7**

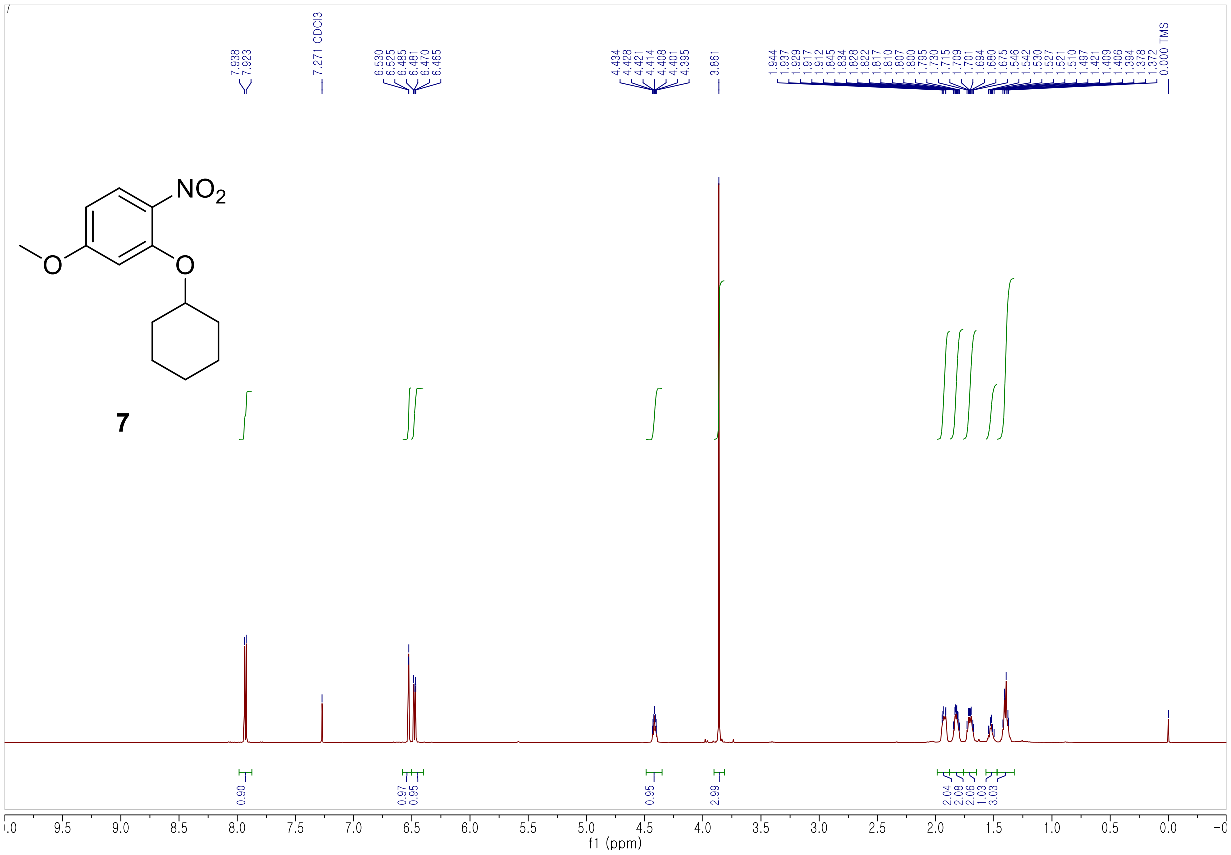

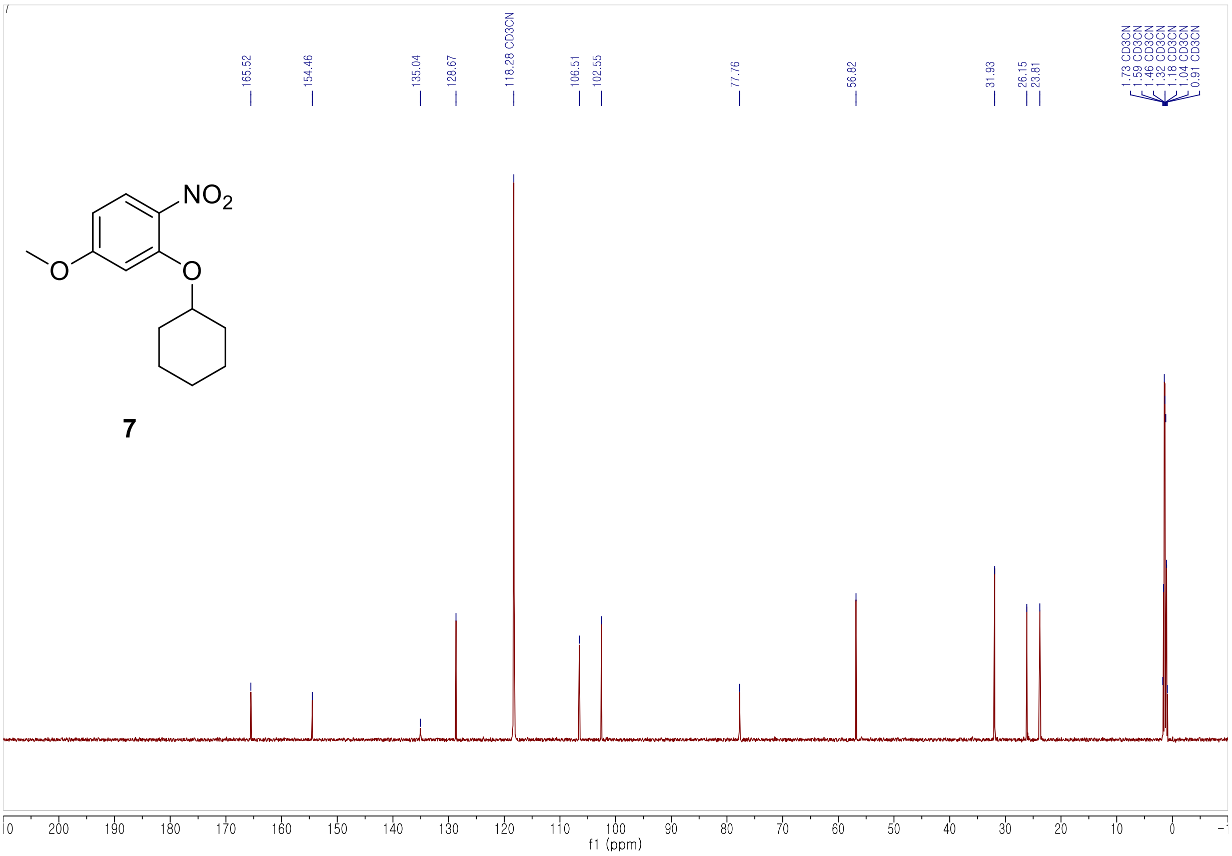

^{1}H NMR (600 MHz, Acetone-d_6) and ^{13}C NMR (150 MHz, Acetone-d_6) spectra of compound **8**

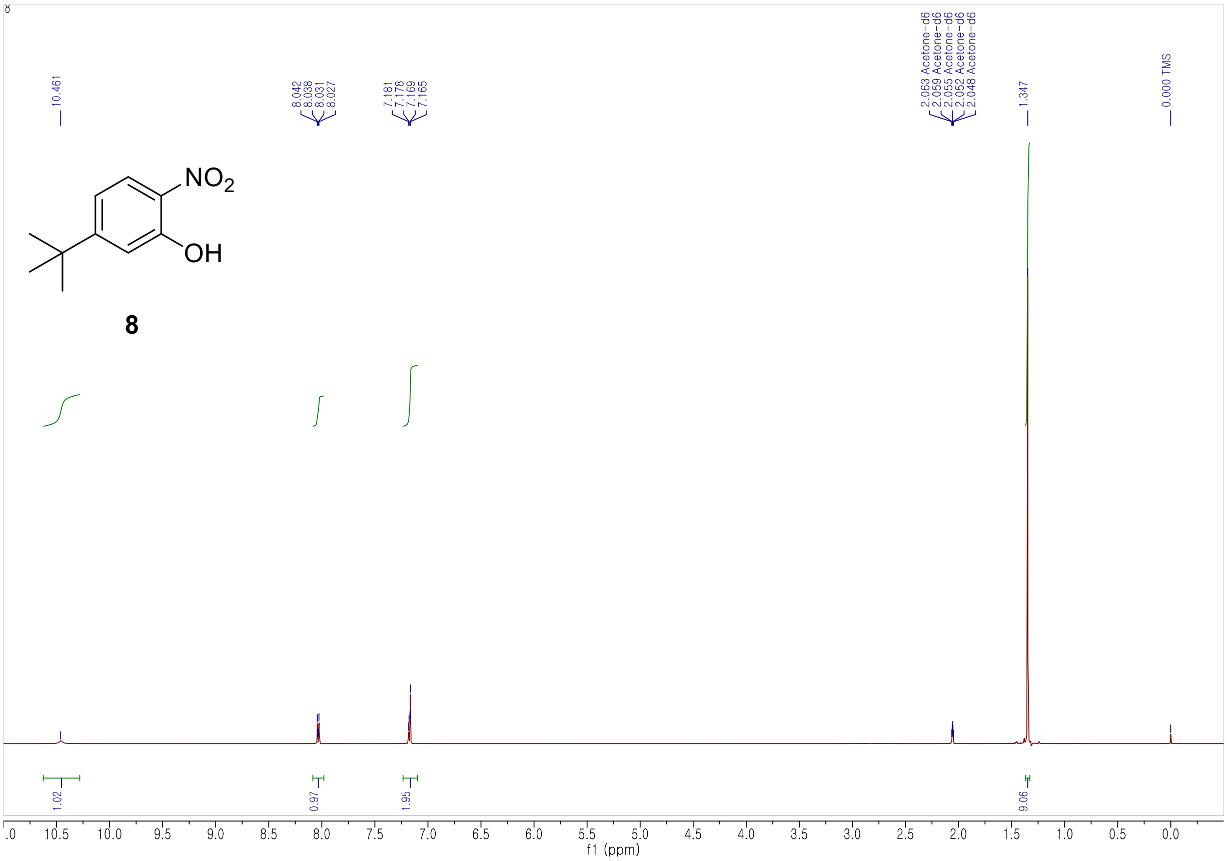

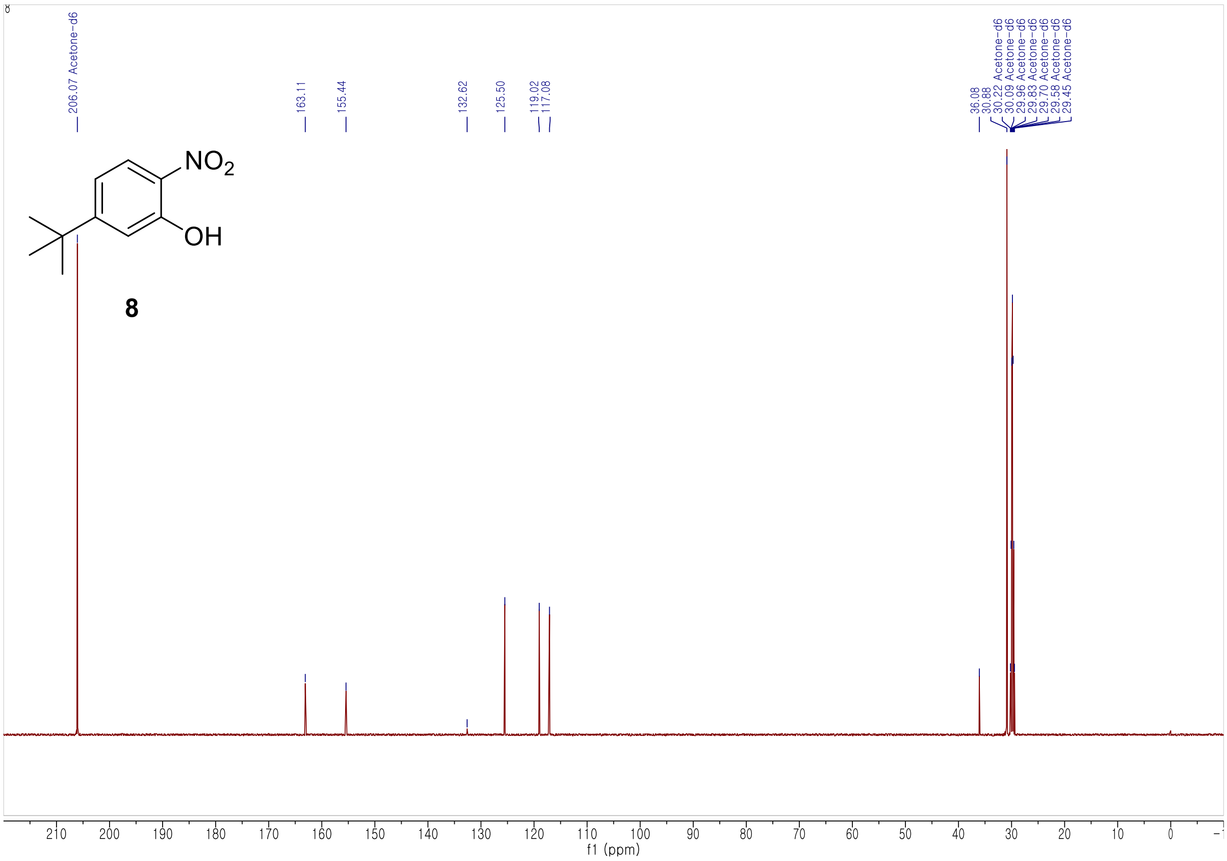

2. Crystallographic data of byproduct 3:

Single crystals of $C_{15}H_{16}N_2O_5$ **[compound 3], [with approximate dimensions** 0.229 × 0.14 × 0.119 mm^3**]**. A suitable crystal was selected and **performed using a SuperNova, Dual, Cu at zero, AtlasS2** diffractometer. The crystal was kept at 99.9(3) K during data collection fitted with an Oxford Cryosystems LN_2 cryostream. Using Olex2 [1], the structure was solved with the ShelXT [2] structure solution program using Intrinsic Phasing and refined with the ShelXL [3] refinement package using Least Squares minimization.

S1 Table. Crystal data and structure refinement for compound 3.

Identification code	compound 3
Empirical formula	$C_{15}H_{16}N_2O_5$
Formula weight	304.30
Temperature/K	99.9(3)
Crystal system	monoclinic
Space group	$P2_1/n$
a/Å	11.29970(10)
b/Å	7.07890(10)
c/Å	18.3378(2)
α/°	90
β/°	104.5330(10)
γ/°	90
Volume/Å^3	1419.90(3)
Z	4
ρ_{calc}g/cm^3	1.423
μ/mm^{-1}	0.909
F(000)	640.0
Crystal size/mm^3	0.229 × 0.14 × 0.119
Radiation	CuKα (λ = 1.54184)
2Θ range for data collection/°	8.364 to 153.144
Index ranges	$-14 \le h \le 14$, $-8 \le k \le 8$, $-23 \le l \le 23$
Reflections collected	30371
Independent reflections	2970 [R_{int} = 0.0240, R_{sigma} = 0.0099]
Data/restraints/parameters	2970/0/202
Goodness-of-fit on F^2	1.048
Final R indexes [I>=2σ (I)]	R_1 = 0.0317, wR_2 = 0.0845
Final R indexes [all data]	R_1 = 0.0333, wR_2 = 0.0860
Largest diff. peak/hole / e Å^{-3}	0.23/-0.28

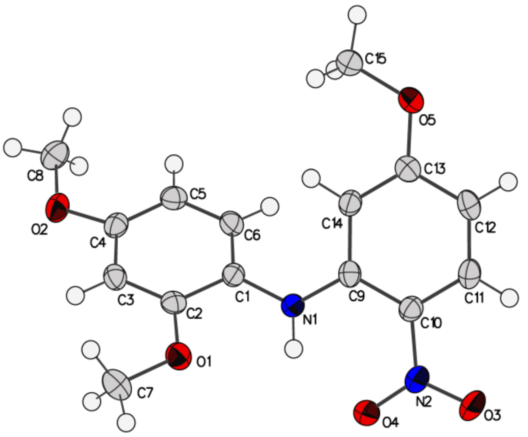

S2 Table. Fractional Atomic Coordinates (×10⁴) and Equivalent Isotropic Displacement Parameters (Å²×10³). U_{eq} is defined as 1/3 of of the trace of the orthogonalised U_{IJ} tensor.

Atom	*x*	*y*	*z*	U(eq)
O1	5862.8(6)	1663.7(11)	6480.9(4)	21.00(17)
O2	3401.4(6)	2727.6(11)	4005.1(4)	22.76(18)
O3	11276.2(7)	210.3(13)	7376.9(5)	39.7(2)
O4	9338.7(6)	-333.7(11)	6977.5(4)	23.24(18)
O5	9997.6(6)	7694.1(10)	5615.6(4)	21.77(17)
N1	7932.0(7)	2012.2(12)	6051.8(5)	18.79(19)
N2	10244.5(8)	701.5(13)	7017.3(5)	21.59(19)
C1	6814.4(9)	2327.7(13)	5510.8(6)	16.8(2)
C2	5722.2(9)	2088.8(13)	5738.3(5)	16.6(2)
C3	4606.7(9)	2255.5(14)	5218.0(6)	18.0(2)
C4	4557.6(9)	2667.8(14)	4465.7(6)	18.0(2)
C5	5625.9(9)	2941.0(14)	4234.3(6)	18.8(2)
C6	6745.9(9)	2734.1(14)	4762.2(6)	18.5(2)
C7	4772.1(10)	1492.6(16)	6739.9(6)	24.0(2)
C8	3269.8(10)	3228.2(17)	3233.1(6)	25.7(2)
C9	8962.8(9)	3078.5(14)	6169.7(5)	16.7(2)
C10	10098.0(9)	2493.8(14)	6648.2(5)	18.1(2)
C11	11133.2(9)	3674.2(15)	6774.4(6)	20.5(2)
C12	11085.0(9)	5393.8(15)	6433.7(6)	20.8(2)
C13	9971.9(9)	5980.5(14)	5941.9(6)	18.5(2)
C14	8941.9(9)	4861.7(14)	5819.3(5)	18.1(2)
C15	8875.3(9)	8332.2(15)	5117.8(6)	23.4(2)

S3 Table. Anisotropic Displacement Parameters (Å^2×10^3). The Anisotropic displacement factor exponent takes the form: $-2\pi^2[h^2a^{*2}U_{11}+2hka^*b^*U_{12}+\ldots]$.

Atom	U_{11}	U_{22}	U_{33}	U_{23}	U_{13}	U_{12}
O1	19.7(3)	24.8(4)	17.8(3)	0.4(3)	3.6(3)	-3.1(3)
O2	16.1(4)	28.9(4)	20.8(4)	2.8(3)	-0.2(3)	0.5(3)
O3	20.1(4)	37.0(5)	52.6(6)	20.1(4)	-8.6(4)	0.4(3)
O4	20.3(4)	21.7(4)	25.7(4)	5.0(3)	2.2(3)	-2.4(3)
O5	19.0(4)	18.6(4)	27.1(4)	2.0(3)	4.7(3)	-3.6(3)
N1	15.7(4)	16.5(4)	22.1(4)	3.7(3)	0.9(3)	-1.5(3)
N2	17.1(4)	23.4(5)	21.7(4)	3.2(3)	0.1(3)	1.4(3)
C1	14.7(4)	12.1(4)	21.5(5)	-0.5(3)	0.8(4)	-1.4(3)
C2	19.5(5)	11.7(4)	18.4(5)	-1.4(3)	4.2(4)	-1.4(3)
C3	15.6(4)	16.1(5)	22.4(5)	-1.6(4)	4.8(4)	-1.2(3)
C4	16.9(5)	13.7(4)	21.3(5)	-1.0(4)	1.0(4)	0.6(3)
C5	21.5(5)	15.9(5)	18.5(5)	1.0(4)	3.9(4)	-1.1(4)
C6	16.8(5)	16.0(5)	22.9(5)	0.3(4)	5.3(4)	-1.9(4)
C7	25.6(5)	26.0(5)	22.1(5)	-4.4(4)	9.6(4)	-7.0(4)
C8	23.5(5)	29.6(6)	20.7(5)	4.2(4)	-0.9(4)	1.0(4)
C9	14.7(4)	17.8(5)	17.4(4)	-2.5(4)	3.4(4)	-0.6(4)
C10	17.2(5)	18.6(5)	17.7(5)	0.5(4)	2.8(4)	0.5(4)
C11	14.7(4)	26.3(5)	19.1(5)	-1.3(4)	1.9(4)	0.1(4)
C12	16.2(5)	24.1(5)	22.0(5)	-3.3(4)	4.4(4)	-5.4(4)
C13	19.7(5)	17.2(5)	19.6(5)	-1.6(4)	6.6(4)	-0.9(4)
C14	15.4(4)	17.7(5)	19.9(5)	-0.1(4)	2.1(4)	0.6(4)
C15	20.9(5)	19.3(5)	28.9(5)	3.8(4)	4.2(4)	-2.1(4)

S4 Table 4. Bond Lengths

Atom	Atom	Length/Å	Atom	Atom	Length/Å	Atom	Atom	Length/Å
O1	C2	1.3644(12)	N1	C1	1.4142(12)	C5	C6	1.3948(14)
O1	C7	1.4328(12)	N1	C9	1.3590(12)	C9	C10	1.4219(13)
O2	C4	1.3667(12)	N2	C10	1.4279(13)	C9	C14	1.4139(14)
O2	C8	1.4305(12)	C1	C2	1.4086(13)	C10	C11	1.4087(14)
O3	N2	1.2365(12)	C1	C6	1.3857(14)	C11	C12	1.3631(15)
O4	N2	1.2460(11)	C2	C3	1.3818(14)	C12	C13	1.4136(14)
O5	C13	1.3561(12)	C3	C4	1.3976(14)	C13	C14	1.3789(13)
O5	C15	1.4368(12)	C4	C5	1.3899(14)			

S5 Table. Bond Angles

Atom	Atom	Atom	Angle/°	Atom	Atom	Atom	Angle/°
C2	O1	C7	117.07(8)	C5	C4	C3	120.53(9)
C4	O2	C8	117.72(8)	C4	C5	C6	118.68(9)
C13	O5	C15	116.64(8)	C1	C6	C5	121.65(9)
C9	N1	C1	127.07(8)	N1	C9	C10	122.75(9)
O3	N2	O4	121.10(9)	N1	C9	C14	120.50(9)
O3	N2	C10	118.97(9)	C14	C9	C10	116.74(9)
O4	N2	C10	119.93(8)	C9	C10	N2	122.04(9)
C2	C1	N1	117.89(9)	C11	C10	N2	117.16(9)
C6	C1	N1	123.08(9)	C11	C10	C9	120.80(9)
C6	C1	C2	118.89(9)	C12	C11	C10	121.23(9)
O1	C2	C1	115.52(9)	C11	C12	C13	118.79(9)
O1	C2	C3	124.44(9)	O5	C13	C12	115.48(9)
C3	C2	C1	120.03(9)	O5	C13	C14	123.54(9)
C2	C3	C4	120.19(9)	C14	C13	C12	120.98(9)
O2	C4	C3	114.30(9)	C13	C14	C9	121.44(9)
O2	C4	C5	125.15(9)				

S6 Table. Torsion Angles.

A	B	C	D	Angle/°	A	B	C	D	Angle/°
O1	C2	C3	C4	-179.00(9)	C3	C4	C5	C6	2.09(15)
O2	C4	C5	C6	-176.49(9)	C4	C5	C6	C1	-2.17(15)
O3	N2	C10	C9	174.19(10)	C6	C1	C2	O1	179.05(8)
O3	N2	C10	C11	-5.55(14)	C6	C1	C2	C3	0.16(14)
O4	N2	C10	C9	-6.26(14)	C7	O1	C2	C1	177.32(8)
O4	N2	C10	C11	174.00(9)	C7	O1	C2	C3	-3.85(14)
O5	C13	C14	C9	179.63(9)	C8	O2	C4	C3	177.14(9)
N1	C1	C2	O1	3.26(13)	C8	O2	C4	C5	-4.21(15)
N1	C1	C2	C3	-175.62(8)	C9	N1	C1	C2	-137.17(10)
N1	C1	C6	C5	176.61(9)	C9	N1	C1	C6	47.23(15)
N1	C9	C10	N2	2.47(15)	C9	C10	C11	C12	-1.07(15)
N1	C9	C10	C11	-177.80(9)	C10	C9	C14	C13	-0.48(14)
N1	C9	C14	C13	178.84(9)	C10	C11	C12	C13	-0.44(15)
N2	C10	C11	C12	178.67(9)	C11	C12	C13	O5	-179.11(9)
C1	N1	C9	C10	-170.19(9)	C11	C12	C13	C14	1.48(15)
C1	N1	C9	C14	10.54(15)	C12	C13	C14	C9	-1.00(15)
C1	C2	C3	C4	-0.22(14)	C14	C9	C10	N2	-178.22(9)
C2	C1	C6	C5	1.05(15)	C14	C9	C10	C11	1.51(14)
C2	C3	C4	O2	177.79(8)	C15	O5	C13	C12	-179.50(9)
C2	C3	C4	C5	-0.93(15)	C15	O5	C13	C14	-0.10(14)

S7 Table. Hydrogen Atom Coordinates (Å×10⁴) and Isotropic Displacement Parameters (Å²×10³) for exp_451.

Atom	*x*	*y*	*z*	U(eq)
H1	7963	1040	6337	23
H3	3887	2093	5369	22
H5	5595	3256	3737	23
H6	7465	2872	4608	22
H7A	4282	478	6477	36
H7B	4318	2651	6645	36
H7C	4986	1235	7271	36
H8A	2418	3247	2975	39
H8B	3687	2317	3000	39
H8C	3616	4457	3206	39
H11	11863	3273	7096	25
H12	11772	6167	6523	25
H14	8218	5289	5499	22
H15A	8628	7460	4707	35
H15B	8997	9559	4925	35
H15C	8252	8404	5389	35

checkCIF/PLATON report

Structure factors have been supplied for datablock(s) Compound_3

THIS REPORT IS FOR GUIDANCE ONLY. IF USED AS PART OF A REVIEW PROCEDURE FOR PUBLICATION, IT SHOULD NOT REPLACE THE EXPERTISE OF AN EXPERIENCED CRYSTALLOGRAPHIC REFEREE.

No syntax errors found. CIF dictionary Interpreting this report

Datablock: Compound_3

```
Bond precision:  C-C = 0.0015 A          Wavelength=1.54184

Cell:            a=11.2997(1)     b=7.0789(1)          c=18.3378(2)
                 alpha=90         beta=104.533(1)      gamma=90
Temperature:     100 K

                 Calculated                  Reported
Volume           1419.90(3)                  1419.90(3)
Space group      P 21/n                      P 1 21/n 1
Hall group       -P 2yn                      -P 2yn
Moiety formula   C15 H16 N2 O5               C15 H16 N2 O5
Sum formula      C15 H16 N2 O5               C15 H16 N2 O5
Mr               304.30                      304.30
Dx,g cm-3        1.423                       1.423
Z                4                           4
Mu (mm-1)        0.909                       0.909
F000             640.0                       640.0
F000'            642.21
h,k,lmax         14,8,23                     14,8,23
Nref             2976                        2970
Tmin,Tmax        0.858,0.897                 0.888,1.000
Tmin'            0.812

Correction method= # Reported T Limits: Tmin=0.888 Tmax=1.000
AbsCorr = MULTI-SCAN

Data completeness= 0.998          Theta(max)= 76.572

R(reflections)= 0.0317( 2797)      wR2(reflections)= 0.0860( 2970)

S = 1.048                  Npar= 202
```

The following ALERTS were generated. Each ALERT has the format
test-name_ALERT_alert-type_alert-level.
Click on the hyperlinks for more details of the test.

Alert level G

```
PLAT007_ALERT_5_G Number of Unrefined Donor-H Atoms ..............           1 Report
PLAT912_ALERT_4_G Missing # of FCF Reflections Above STh/L=  0.600          7 Note
PLAT978_ALERT_2_G Number C-C Bonds with Positive Residual Density.        14 Note
```

```
   0 ALERT level A = Most likely a serious problem - resolve or explain
   0 ALERT level B = A potentially serious problem, consider carefully
   0 ALERT level C = Check. Ensure it is not caused by an omission or oversight
   3 ALERT level G = General information/check it is not something unexpected

   0 ALERT type 1 CIF construction/syntax error, inconsistent or missing data
   1 ALERT type 2 Indicator that the structure model may be wrong or deficient
   0 ALERT type 3 Indicator that the structure quality may be low
   1 ALERT type 4 Improvement, methodology, query or suggestion
   1 ALERT type 5 Informative message, check
```

It is advisable to attempt to resolve as many as possible of the alerts in all categories. Often the minor alerts point to easily fixed oversights, errors and omissions in your CIF or refinement strategy, so attention to these fine details can be worthwhile. In order to resolve some of the more serious problems it may be necessary to carry out additional measurements or structure refinements. However, the purpose of your study may justify the reported deviations and the more serious of these should normally be commented upon in the discussion or experimental section of a paper or in the "special_details" fields of the CIF. checkCIF was carefully designed to identify outliers and unusual parameters, but every test has its limitations and alerts that are not important in a particular case may appear. Conversely, the absence of alerts does not guarantee there are no aspects of the results needing attention. It is up to the individual to critically assess their own results and, if necessary, seek expert advice.

Publication of your CIF in IUCr journals

A basic structural check has been run on your CIF. These basic checks will be run on all CIFs submitted for publication in IUCr journals (*Acta Crystallographica*, *Journal of Applied Crystallography*, *Journal of Synchrotron Radiation*); however, if you intend to submit to *Acta Crystallographica Section C* or *E* or *IUCrData*, you should make sure that full publication checks are run on the final version of your CIF prior to submission.

Publication of your CIF in other journals

Please refer to the *Notes for Authors* of the relevant journal for any special instructions relating to CIF submission.

PLATON version of 27/03/2017; check.def file version of 24/03/2017

Datablock Compound_3 - ellipsoid plot

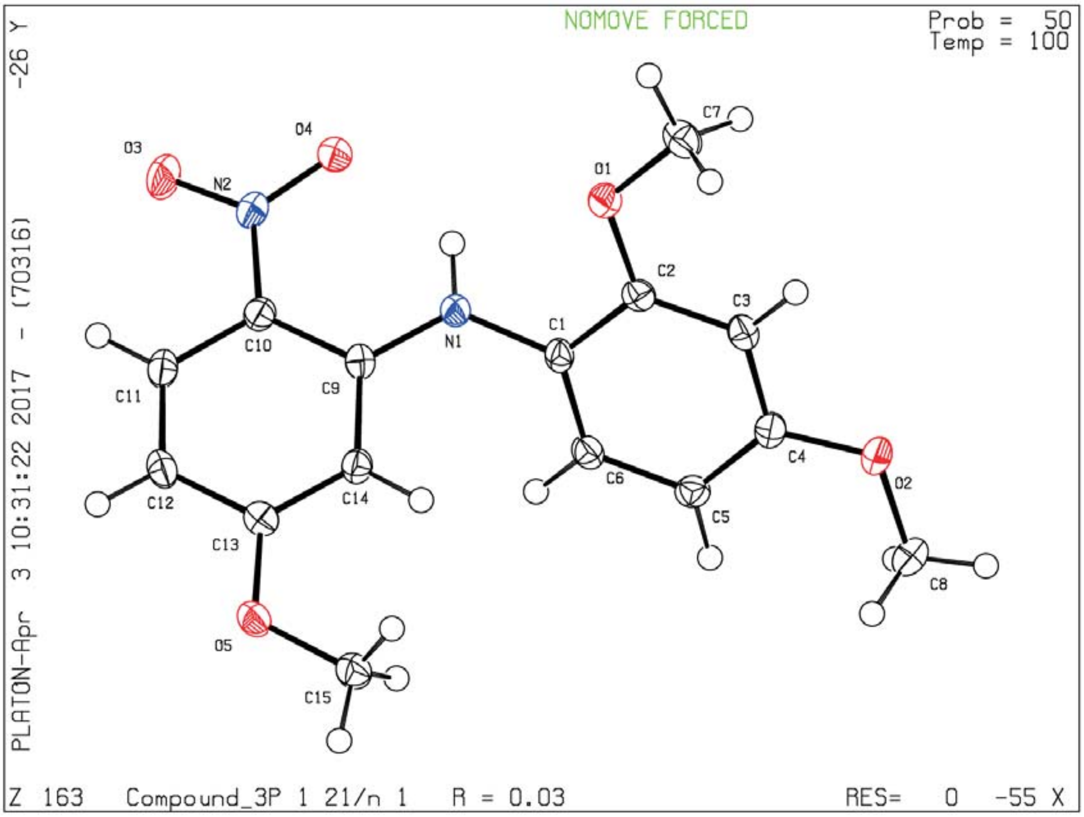

References

1. Dolomanov OV, Bourhis LJ, Gildea RJ, Howard JAK, Puschmann, H. OLEX2: a complete structure solution, refinement and analysis program. J Appl Crystallogr 2009(42), 339-341.
2. Sheldrick GM. SHELXT– Integrated space-group and crystal-structure determination. Acta Crystallogr A Found Adv 2015(71), 3-8.
3. Sheldrick GM. Crystal structure refinement with SHELXL. Acta Crystallogr C Struct Chem. 2015(71), 3-8.
